# Socioeconomic disparities in the postnatal growth of preterm infants: a systematic review

**DOI:** 10.1038/s41390-024-03384-0

**Published:** 2024-07-18

**Authors:** Krithi Ravi, Aneurin Young, R. Mark Beattie, Mark J. Johnson

**Affiliations:** 1 Department of Anaesthesia, Royal Alexandra Hospital, NHS Greater Glasgow and Clyde, Paisley, UK; 2 Department of Neonatal Medicine, Princess Anne Hospital, University Hospital Southampton NHS Foundation Trust, Southampton, UK; 3 NIHR Southampton Biomedical Research Centre, University Hospital Southampton NHS Foundation Trust and University of Southampton, Southampton, UK; 4 Department of Paediatric Gastroenterology, Southampton Children’s Hospital, University Hospital Southampton NHS Foundation Trust, Southampton, UK

## Abstract

**Objective:**

To determine the effect of parental socioeconomic status (SES) on the postnatal growth of preterm infants.

**Methods:**

A systematic review (PROSPERO registration CRD42020225714) of original articles from Medline, Embase, CINAHL Plus and Web of Science published 1946-2023 was undertaken. Studies were included if they reported anthropometric growth outcomes for preterm infants according to parental SES. Data extraction and assessments of bias and health equity impact were conducted using custom-designed forms.

**Results:**

A narrative synthesis of twelve included studies was performed. Most infants were moderate to late preterm. The settings, growth outcomes, timings of growth measurement, and SES measures were heterogenous. Six studies demonstrated an adverse effect of low parental SES on the extrauterine growth of preterm infants, five studies showed no effect, and one study showed a potentially beneficial effect. All studies had a high risk of bias, especially confounding and selection bias. The health equity impact of included studies was largely negative.

**Conclusion:**

Limited and low-quality evidence suggests that socioeconomic minoritisation may adversely impact the growth of preterm infants, thereby widening existing socioeconomic health inequities. Observational studies informed by theorisation of the mechanistic pathways linking socioeconomic minoritisation to adverse postnatal growth are required to identify targets for intervention.

**Impact:**

- Limited evidence suggests low parental socioeconomic status (SES) adversely affects the postnatal growth of preterm infants across different settings.
- Early growth of preterm infants predicts neurodevelopmental outcomes and the risk of cardiovascular and metabolic disease in adulthood.
- Systematic screening of over 15,000 articles identified only twelve studies which reported postnatal growth outcomes for preterm infants according to parental SES.
- The health equity impact of the included studies was systematically assessed, and found to be negative overall.
- This study highlights limitations in existing evidence on the association between parental SES and postnatal growth, and delineates avenues for future research.

## Introduction

Socioeconomic deprivation has adverse health consequences throughout the life course. It is associated with adverse pregnancy outcomes, greater infant and childhood mortality, poorer development in childhood, a shorter life expectancy and poorer health-related quality of life^1–4^.

The deleterious impact of socioeconomic deprivation on maternal and child health outcomes in the perinatal period can lead to the intergenerational transfer of morbidity, perpetuating health disparities. Low socioeconomic status (SES) is a risk factor for preterm birth^1,4^ and intrauterine growth restriction^5^ across the world. Thus, by the time of birth, socioeconomic minoritisation is already embodied in infants due to poorer growth in-utero and prematurity.

The extrauterine growth of preterm infants has been extensively investigated, as it has implications for both childhood development and risk of adult disease. Early postnatal growth in preterm infants is an important predictor of later neurodevelopment outcomes^6,7^. On the other hand, accelerated growth following preterm birth is associated with later cardiovascular and metabolic disease^8,9^. The postnatal weight loss consistently observed in previous studies of preterm infants^10,11^ has been shown to be avoidable with the standardised implementation of improved neonatal guidelines^12^.

Eliciting the impact of social circumstances on outcomes of preterm infants has been identified as a key priority for collaborative research^13^. Considering the significant impact of preterm infants’ postnatal growth pattern on their long-term health, and the fact that preterm birth occurs disproportionately in those who are socioeconomically deprived, the effect of SES on the extrauterine growth of preterm infants needs to be delineated. We conducted a systematic literature review to investigate the impact of parental SES on postnatal growth outcomes of preterm infants.

## Methods

This study adheres to the Preferred Reporting Items for Systematic Reviews and Meta-Analyses (PRISMA) guidelines (refer to the PRISMA Checklist). Search terms, eligibility criteria, and proposed data synthesis methods were registered with PROSPERO (CRD42020225714) prior to the start of the review. We included original research studies reporting data from humans, including clinical trials and observational studies, which included preterm infants born at less than 37 weeks’ gestational age. We only included studies which reported a measure of parental SES as an exposure and reported anthropometric infant growth outcomes according to parental SES. The eligibility criteria are summarised in Table S1. The initial inclusion criteria specified English language articles published between 1990 and 2020, but the inclusion criteria were later amended to original research studies in all languages published between 1946 and 2023, including observational studies and clinical trials. The reference lists of relevant reviews were searched to identify additional eligible articles.

### Searches and information sources

A literature search was conducted using the search strategy detailed in Table S2. The electronic databases MEDLINE (1946 onwards) and Embase (1947 onwards), CINAHL Complete and the Web of Science website (wok.mimas.ac.uk, 1970 onwards) were searched for published literature and conference proceedings. No date or language restrictions were applied. Due to the lack of availability of translators, Google Translate (translate.google.com) was used for non-English language articles. The last search was run on 04 February 2023.

### Study selection

Titles and abstracts were manually screened, and full texts were screened where the article’s eligibility for inclusion remained unclear. No automation tools were used in the article selection process. The full text of any potentially eligible article was then reassessed against the selection criteria and included or excluded as appropriate. Two authors (K.R. and A.Y.) independently screened a subset 1037 titles and abstracts; conflicts were resolved through discussion to ensure consistent application of the eligibility criteria. A single author (K.R.) conducted the remainder of title and abstract screening, and full text screening.

### Data collection

Data extraction was conducted by a single author (K.R.) using a purpose-built data collection form (Table S3).

### Assessment of risk of bias

Risk of bias within studies was conducted by a single author (K.R.) by assessing the study design and methodology using a custom-designed table, adapted from the CASP checklists^14^ and the framework developed by Viswanathan et al.^15^ for observational studies (Table S4). Tools designed to assess the risk of bias for Randomised Controlled Trials (RCTs) were not used as no RCTs were included.

### Data synthesis

Growth outcomes for preterm infants according to parental SES were extracted and assessed for suitability for meta-analysis.

## Results

**Fig. 1:**
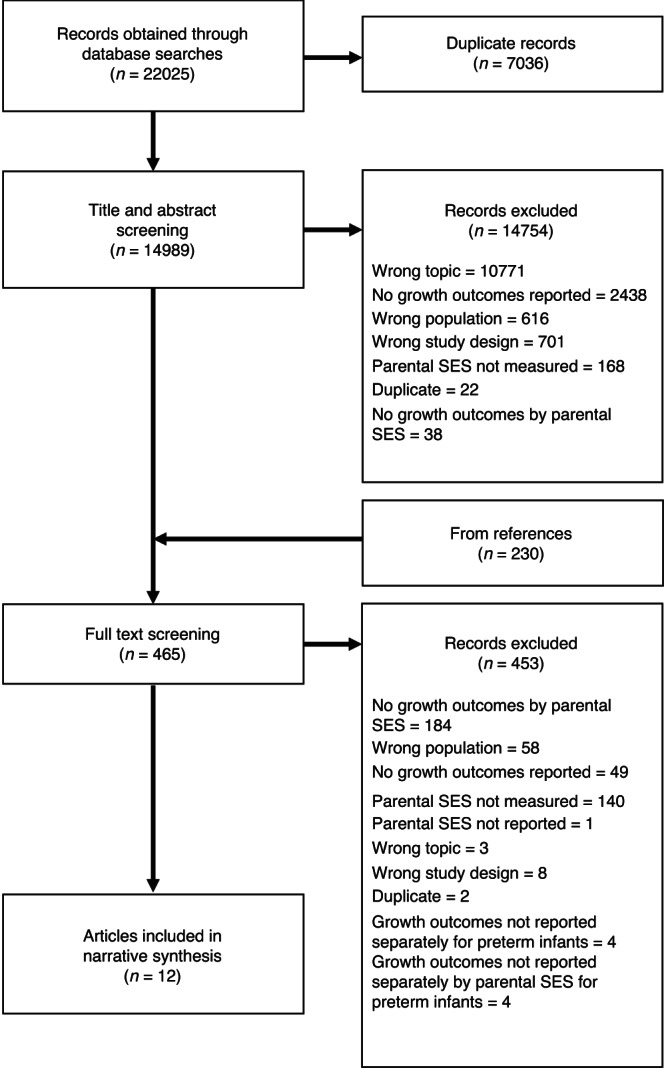
Preferred Reporting Items for Systematic reviews and Meta-Analyses (PRISMA) flowchart. Flow chart depicting article screening process and flow of articles from initial search identification through to selection or rejection.

Twelve out of 15,219 articles assessed were included (Fig. 1): Ahn^16^, Bocca-Tjeertes^17^, Sammy^18^, Teranishi^19^, Holmqvist^20^, Ghods^21^, Ni^22^, Kelleher^23^, Fu^24^, Liang^25^, Sices^26^ and Peterson^27^. Seventeen articles reported some measure of parental SES and growth outcomes for preterm infants but were excluded because growth data were not reported separately according to parental SES (e.g. only the regression or correlation coefficient or a test statistic was reported, or the authors mentioned a lack of association between parental SES and growth outcomes without presenting data). A summary of these articles is presented in Table S5. The authors of these studies were not contacted to provide raw data.

Characteristics of included studies are summarised in Table 1. Seven of the included studies^16,18–20,22–24^analysed data collected prospectively for a cohort of children, whereas three studies^21,25,26^ analysed retrospectively collected data, one study^17^analysed a mixture of retrospectively collected and cross-sectional data for the same cohort of children, and one study^27^analysed data collected prospectively and retrospectively from cohorts. Nine studies were conducted in high-income countries (South Korea, United States, United Kingdom, the Netherlands, Austria and Sweden), two studies in an upper middle-income country (China), and one study in a lower middle-income country (Kenya). Six studies had mainly moderate to late preterm infants^16–18,20,23,24^, whereas five studies had mainly very and extremely preterm infants^21,22,25–27^. Most infants did not have congenital anomalies or syndromes.

**Table 1:** Summary of study characteristics.

Reference	Setting	Study design	Population	Exclusion criteria	Measure of parental socioeconomic status	Growth outcomes measured	Growth outcomes for preterm infants reported according to parental socioeconomic status
**Ahn** ^16^	**South Korea:** Inborn infants at a University hospital in Incheon.	Secondary analysis of prospectively collected growth data for a cohort of preterm infants.	**Preterm infants born after 32 weeks’ gestation**. ***n*** = **238**. Mean GA ^36^34.8 weeks (1.1). Range GA: 32–-36 + 6 weeks.	Outborn infants, “socially vulnerable” infants (e.g. those with unmarried mothers, adopted infants), infants with congenital malformations that affect physical growth.	**Maternal employment status: Yes/No**. Methods for ascertaining maternal employment status not reported.	**Weight, length and head circumference measured from birth to 6 months corrected age**. Measurements were taken directly either during NICU admission or at outpatient follow-up. Where direct measurement was not possible, growth data were collected from the records.	1. Effect of maternal employment status on variation in weight, length and head circumference growth by 6 months corrected age analysed using a generalized linear model and reported. 2. Graphs of weight gain from birth to 6 months corrected age shown separately for infants of employed and unemployed mothers.
**Bocca-Tjeertes** ^17^	**Netherlands:** Community-based sample of participants recruited at a routine 4-year well-child visit at Preventive Child Health Care Services (PCHCs).	Secondary analysis of a subgroup of the Lollypop cohort study, with data collected retrospectively from medical records from birth to 4 years, and additional measurements taken at 4 years. The Lollypop study collected data on the growth, development and general health of preterm infants.	**Early and moderately preterm infants born before 36 weeks’ gestation**. ***n*** = **1123** (infants with growth data available). Mean GA: 34 weeks. Range GA: 32 – 35 + 6 weeks.	Infants with congenital malformations and syndromes, infants for who gestational age was not verified “beyond reasonable doubt”.	**Maternal education level: low, moderate/high (definitions not reported)**. 2. **Family income: low, moderate/high (definitions not reported)**. Maternal education level and family income ascertained through a parental questionnaire administered at the 4-year well-child visit.	**Weight and height measurements from birth to 4 years, and head circumference measurements from birth to 12 months**. Measurements were obtained from hospital records and records of routine well-child visits at the PCHCs. SD scores for weight at birth, height and head circumference at birth were calculated using the Kloosterman ^92^and Usher and McLean ^93^curves respectively. Growth restraint was defined by measurements 2 SD scores below the median growth of the Dutch population, deduced from the fourth nationwide Dutch growth survey with measurements of children born shortly before 1997.	Postnatal growth restraint at 4 years (for weight and height) and 1 year (for head circumference) reported separately according to maternal education level and family income.
**Sammy** ^18^	**Kenya:** Preterm infants born at the county referral hospital for the former Kitui and Mwingi districts.	Prospective cohort study of preterm infants discharged from the Kitui District Hospital newborn unit, followed up 2 weeks post-discharge.	**Preterm infants born at less than 37 weeks’ gestation and discharged from the newborn unit between April and June 2014**. ***n*** = **106** (infants with growth data at 2 weeks). Mean GA and range of GA not calculable as lower limit of gestational age not reported. More than 50% of infants born between 33 and 36 weeks.	Infants with congenital anomalies likely to interfere with their growth, or with guardians declining to participate in the study.	**Maternal education level: no education, primary education, secondary education and tertiary education**. Information on maternal education obtained via a questionnaire-guided interview with guardians at discharge.	**Weight, length and head circumference were measured at discharge from the newborn unit, and at 2 weeks post-discharge**. Length of hospital stay varied from 2 to 30 days, and therefore time of follow-up was varied.	Number of infants with “optimal growth” (not defined) and “growth deficit” (not defined) reported according to maternal education level.
**Teranishi** ^19^	**United Kingdom:** children born across England, Scotland and Wales.	Birth cohort study, with a wide range of data collected from parental interviews, medical examinations, school questionnaires, personal questionnaires and attainment tests.	**White singleton infants born 3**–**9 Mar 1958 who had perinatal data and data at 23 years available**. Preterm birth was considered a dichotomous variable (GA < 37 weeks vs ≥ 37 weeks). Number of preterm infants unknown. Mean GA and range of GA not calculable.	Unknown.	**Social class coded by father’s occupation according to the Registrar General’s Classification (I: professionals, II: intermediate, IIINM: skilled non-manual, IIIM: skilled manual, IV: partly manual, V: unskilled manual)**. Questionnaire administered to parents at time of birth used to ascertain father’s occupation.	**Height was measured at 7, 11 and 16 years. Height was self-reported at 23 years**. Height deficit was defined as the difference in height between low birthweight (LBW, birthweight < 2500 g) and appropriate birthweight (ABW, birthweight ≥ 2500 g) children calculated (mean height of ABW children – mean height of LBW children.)	Graphs of height deficit at ages 7, 11, 16 and 23 are shown separately for preterm infants in social classes I and II and those in classes IV and V.
**Holmqvist** ^20^	**Sweden:** preterm infants born at a University hospital in Lund.	Prospective cohort study of preterm infants followed up until 48 months of age.	**Low risk, inborn preterm infants (GA** < **37) born Jan-Dec 1979**: vaginal delivery, singleton pregnancy, cephalic presentation, preterm labour of unknown aetiology, no additional maternal complications (e.g. maternal diseases, signs of preeclampsia, anaemia or vaginal bleeding), no additional foetal complications (biparietal diameter within 2 SD, “normal” symphysis-fundus height). ***n*** = **35**. Mean GA = 34 weeks.	Maternal complications (e.g. maternal diseases, signs of preeclampsia, anaemia or vaginal bleeding), foetal complications (biparietal diameter outside 2 SD, symphysis-fundus height not considered “normal”).	**Maternal education: well educated mothers (> 11 school years), less well educated mothers (< 11 school years)**. Questionnaire administered at one of the follow-up visits (timing not specified).	**Weight, length and head circumference measured at birth, and at 3, 7, 12, 24 and 48 months**. Weekly individual growth increments for weight, length and head circumference calculated by dividing difference between 2 consecutive measurements by the number of weeks in between for 5 time periods: birth to 3 months, 3-7 months, 7-12 months, 1-2 years, 2-4 years.	Weekly growth increments in weight, height and head circumference for each timer period compared between infants with well educated mothers and infants with less well educated mothers.
**Ghods** ^21^	**Austria:** preterm infants admitted to a NICU at a University hospital in Vienna.	Retrospective cohort study of preterm infants followed up until 66 months of age.	**All preterm (GA** < **37) or VLBW (birthweight** < 1500 **g) infants admitted to the NICU born between 2000 and 2002.** ***n*** = **173**. Mean GA of infants with head circumference (HC) catch-up ^92^ = 28.04 weeks (2.6 weeks). Mean GA of infants without HC catch-up ^92^ = 29.53 weeks (2.3 weeks).	Infants lost to follow-up, infants whose medical records were incomplete, infants whose head circumference was not measured and could not be interpolated at 1 year corrected age.	**Maternal education** ≥ **12 years:** Yes/No. 2. **Home Facilities: Good, Adequate, Inadequate**. Assessed based on indirect questioning and assessment of home environment by the interviewer (e.g. light, ventilation, accommodation). 3 **Financial Situation: Good, Enough, Inadequate**. Estimated based on education level (parent not specified), occupation and social facilities.	**Weight, height/length and head circumference (HC) were measured at birth, during the NICU stay and at 3, 6, 9, 12, 24, 40, 54 and 66 months corrected age**. Z-scores for measurements taken during the NICU stay were calculated using the data from Fenton et al^94^. Z-scores for measurements taken after discharge were calculated using the WHO 2006 growth standards^95^. HC catch-up defined as an increase of 0.67 Z-score units between birth and 1 year corrected age.	Parental socioeconomic characteristics compared between infants with and without HC catch-up.
**Ni** ^22^	**United Kingdom:** preterm infants born across the UK and Ireland.	Prospective cohort study of a birth cohort of preterm infants followed up until 19 years of age.	**Preterm infants born at** < **26 weeks’ gestation between Mar and Dec 1995 and enrolled in the EPICure study who were alive at 16 years and consented to assessment at 19 years.** ***n*** = **129**. Mean GA of infants with metabolic syndrome at 19 years ^92^ = 24.95 weeks (0.78 weeks). Mean GA of infants without metabolic syndrome at 19 years ^92^ = 24.76 weeks (1.21 weeks).	Participants with data missing for BMI and central systolic BP at 19 years, participants who died or were lost to follow-up before assessment at 19 years.	1. **Maternal education: A level or above, GCSE or below**. 2. **Parental occupation: Higher (non-manual employment), Lower (manual employment or unemployed)**. Parent questionnaire administered when children were 2.5 years old.	**Weight was measured at birth, 40 weeks postmenstrual age, and at 2.5, 6, 11 and 19 years. Height was measured at 19 years. Waist and hip circumferences were measured at 19 years**. Z- scores for weight were calculated at each age based on UK population norms. Change in weight z-scores were calculated for birth to term, term to 2.5 years, 2.5 to 6 years and 6 to 11 years. BMI at 19 years was calculated. The waist:hip ratio was calculated at 19 years.	BMI distribution at 19 years is plotted separately for participants with lower (occupation-based) parental socioeconomic status and higher parental socioeconomic status.
**Liang** ^25^	**China**: Preterm infants born at a teaching hospital in Nantong (Jiansu province).	Retrospective cohort study of preterm infants followed up until 18 months, some of whom received the intervention of family integrated care (FIcare group) and some of whom received NICU traditional care (control group) during the NICU admission.	**Preterm infants born at** > **28 weeks’ and** < 34 weeks’ gestation between Jan 2018 and Sep 2020, with birthweight 1000 – 2499 g, admitted to the NICU within 24 **hours after birth, with parents possessing basic reading and comprehension skills and the ability to care for their infant.** ***n*** = **215**. Mean GA for infants in FIcare group ^92^ = 30.03 weeks (1.38 weeks). Mean GA for infants in control group ^92^ = 29.93 weeks (1.30 weeks).	Infants with congenital genetic metabolic diseases, digestive tract malformations, severe congenital heart disease, central nervous system and endocrine diseases, or other severe congenital growth and development abnormalities; infants who received invasive respiratory therapy; infants who required surgery; infants who received comfort treatment; and infants with parents who had previous mental health history.	1. **Primary caregiver education: below junior school, high school and technical secondary school, college degree or above**. 2. **Average monthly household income:** < **3000 CNY, 3000 – 5000 CNY**, > **5000 CNY**. Questionnaire administered to parents by researchers (timing and setting of administration not specified).	**Weight, length and head circumference measured at 1, 3, 6, 12 and 18 months**.	Weight, length and head circumference of infants at 12 months are reported according to average monthly household income, and compared between infants of households in different monthly income categories.
**Fu** ^24^	**China:** Preterm infants born across Jianxing (Zheijang province).	Prospective cohort study of a birth cohort of children followed up until school age.	**Singleton infants born before 37 weeks’ gestation and enrolled in the Jianxing Birth Cohort between 1999 and 2013. n** = **2125**. Median GA = 36 weeks (IQR 35 – 36 weeks)	Mother-child pairs without complete data for the detection of childhood overweight/obesity at 4 – 7 years corrected age, or missing data for any variables considered in the study.	1. **Maternal education: <** **High school, High school, >** **High school**. 2. **Maternal occupation: Farm work/housework, Routine job, Temporary work, Others**. Questionnaire administered to mothers in local clinics via interview during enrolment in the cohort and during pregnancy.	**BMI was calculated at 4 – 7 years corrected age**. BMI z-scores were calculated at corrected ages between 4 and 5 years using the 2006 WHO Child Growth Standards^96^; overweight and obesity were defined as BMI z-scores between 2 and 3, and > 3 respectively. The 2007 WHO Child Growth Standards ^97^were used for children older than 5 years corrected age; overweight and obesity were defined as BMI z-scores between 1 and 2, and > 2 respectively Although not specified by the authors, height and weight were presumably measured at 4 – 7 years corrected age.	Numbers of children with and without obesity at 4 – 7 years corrected age (mean age 6.8 years +/− 0.9 years) reported for each maternal occupation and maternal education category. Distributions of maternal occupation and maternal education were compared between infants with and without overweight/obesity at 4 – 7 years corrected age.
**Sices** ^26^	**United States:** Preterm infants born at a teaching hospital in Cleveland.	Retrospective analysis of growth and developmental data from a cohort of extremely low birthweight infants followed to 20 months corrected age.	**Infants with birthweight 501 – 1000** **g born between 1997 and 1999, and admitted to the NICU, who survived until 20 months corrected age. n** = **154**. Mean GA ^92^ = 25.9 weeks (1.8 weeks).	Infants with congenital malformations known to have a direct impact on growth, infants without a minimum of two consecutive growth measurement points, infants discharged after the age of 49 weeks.	**Maternal education less than high school:** Yes/No.	**Weight, length and head circumference (HC) were measured at clinic visits at 40 weeks corrected age (39.2** + **/**− **3.0 weeks), and at 4 (4.4** + **/**− **0.7), 8 (8.6** + **/**− **1.0) and 20 months (19.0** + **/**− **1.2) months corrected age**. Weight z-scores were calculated at birth and at 40 weeks using the sex-specific standards of Kramer et al^98^. At birth and 40 weeks, length and HC z-scores were calculated using the Usher and McLean curves^93^. At 4, 8 and 20 months corrected age, and for infants older than 43 weeks at the first post-discharge follow-up, weight, length and HC z-scores were calculated from US CDC sex-specific normative data^99^. Growth failure defined as a decrease in weight z-score of over 0.67 during any of the three study periods: 40 weeks to 4 months, 4 to 8 months, and 8 to 20 months.	Number of infants with growth failure in each period and the number without growth failure in any period are reported for mothers with less than high school education (and are deducible for mothers with high school education or greater). Distribution of maternal education compared between infants with growth failure in a period, and infants without growth failure in any period.
**Peterson** ^27^	**United States:** Preterm infants born at a teaching hospital in Cleveland and two other tertiary centres.	Analysis of data from two cohorts of very low birthweight infants: 1) recruited at birth and followed up prospectively until school age, 2) recruited at early school age with retrospectively collected perinatal data.	**Infants with birthweight** < 750 g born between 1982 and 1986 and a matched group of infants with birthweight 750–1499 **g from the same hospital and with the same sex, race and birth date within 3 months.** ***n*** = **128**. Mean GA ^92^ = 27.6 weeks (2.8 weeks).	Missing or unreliable head circumference measurement at school age.	**Maternal education less than high school:** Yes/No.	**Height, weight and head circumference (HC) measured at the school age follow-up at a mean age of 6.8 years (+/**− **0.9 years)**. HC z-score at school age was calculated using Roche et al’s reference data ^100^. Subnormal HC was defined as a measurement more than 2 SD below the mean for age.	Numbers of infants with and without subnormal HC at school age reported separately for mothers with less than high school education (and are deducible for mothers with high school education or greater). Distribution of maternal education compared between infants with and without subnormal HC at school age.
**Kelleher** ^23^	**United States:** Preterm infants born at 8 medical centres associated with medical schools across the US.	Analysis of data from the Infant Health and Development Program (IHDP), a national multicentre randomised controlled trial to investigate the effect of early intervention on the cognitive, behavioural and health status of preterm low birthweight infants followed from birth until 3 years of age.	**All infants enrolled in the IHDP. Infants born between Jan-Oct 1985 at the 8 participating sites at** ≤ **37 weeks’ gestation with birthweight** ≤ 2500 **g were eligible for the IHDP.** ***n*** = **771**. Mean GA according to failure to thrive (FTT) case status: FTT infants: (*n* = 180) = 33 weeks. Non-FTT infants: (*n* = 591) = 33.1 weeks.	Infants who lived outside of the catchment area, infants discharged outside the recruitment time period, infants who died within the first 48 hours of life, all triplets, all quadruplets, and twins of ineligible children. Only one infant from each pair of eligible twins was included. Maternal drug or alcohol abuse, maternal inability to communicate adequately in English, maternal report of psychiatric hospitalisation. Hospitalisation longer than 60 days after 40 weeks corrected age, oxygen support for more than 90 days, severe neurologic abnormality, severe sensory deficit or chromosome-multiple anomaly syndrome. Lack of parental consent, or family refusal of group assignment. Infants lost to follow-up before 30 months. Infants who met some but not all of the criteria for failure to thrive (see “Growth outcomes measured” column).	1. **Maternal education: < High school, High school graduate, Some college, ≥ College graduate**. 2. **(Annual) family income:** < **$10,000, $10-20,000**, > **$20,000**. Data gathered via a family interview by a nurse clinician during the newborn’s nursery stay. It is not specified whether mothers directly reported their education level.	**Weight, height and head circumference measured at the follow-up clinics at 40 weeks corrected age, and 4, 8, 12, 18, 24, 30 and 36 months corrected age**. FTT defined as: a) Infants coded by the developmental clinician during a health assessment as having FTT and b) Weight < 5^th^ percentile at ≥ 2 points in time and c) Weight growth during the preceding months was less than average for sex and corrected age as determined by velocity growth curves. Infants who met criteria b) and c) whose growth curves were determined to represent FTT upon blind review by 2 developmental paediatricians were also coded as FTT.	Percentages of infants with and without FTT reported according to maternal education level. Distribution of maternal education compared between infants with and without FTT. Logistic regression analysis conducted to explore the association between maternal education and risk of FTT, adjusting for small for gestational age status at birth, abnormal or suspect neurologic exam at birth, birthweight, maternal age, maternal height and whether or not the infant was living with their father.

Due to the heterogeneity of growth and parental SES measures across studies, we were unable to perform a meta-analysis and instead conducted a narrative synthesis of the findings^28^. Ten studies considered maternal or primary caregiver education a measure of parental SES^17,18,20–27^. Three studies considered family income^17,23,25^. Three studies used parental occupational class as a proxy for SES^19,22,24^. In the study by Ghods et al., a psychologist interviewing the parents also rated families’ Home Facilities, and estimated a composite Financial Situation measure. Interestingly, Ahn et al. considered maternal employment to be a sociocultural factor due to insufficient support for maternity leave and childcare for employed mothers (Table 1).

### Study findings

Seven studies demonstrated an effect of SES on the postnatal growth of preterm infants. Findings are summarised in Table 2.

**Table 2:** Summary of study findings.

Study	Relationship between parental socioeconomic status and growth of preterm infants			
**Ahn** ^16^	Weight gain of infants born to employed mothers was lower than that of infants born to unemployed mothers from birth to 6 months corrected age. • Weight gain velocity diverged between infants of employed and unemployed mothers at 3 months, resulting in a maximum weight difference of approximately 900 g at 5 months. • Weight gain velocity of infants of employed mothers increased between 5 and 6 months of age, resulting in a narrowed weight difference of approximately 400 g at 6 months. There was a significant difference in weight gain over time between infants of employed mothers and infants of unemployed mothers (*p* < 0.001). There was no significant difference in change in length or head circumference over time between the 2 groups.			
**Bocca-Tjeertes** ^17^	In univariate analyses, a low level of maternal education, but not low family income, was associated with head circumference growth restraint compared with the median population growth at 1 year. • The Odds Ratio of head circumference growth restraint for infants of mothers with a low education level compared to a high education level was 5.3 (95% CI 1.4 –20.8) in the multivariable logistic regression adjusting for gestational age, family income, smoking during pregnancy, maternal age, conception by IVF or ICSI, infant sex, multiple pregnancy, and breastfeeding during the first 6 months of life.			
**Sammy** ^18^	Maternal education level did not have a significant effect on the likelihood of growth deficit at 2 weeks following discharge from the neonatal unit. • Simple linear regression analysis showed no statistically significant difference in the odds of growth deficit in infants born to mothers with primary, secondary and tertiary level education compared to infants born to mothers with no education.			
**Teranishi** ^19^	At all time points considered (7, 11, 16 and 23 years), the height deficit between low birthweight (LBW, < 2500 g) and appropriate birthweight (ABW, ≥ 2500 g) infants was lower in preterm infants in social classes I & II (fathers in professional and intermediate occupations) than those in social classes IV & V (fathers in partly manual and unskilled manual occupations). • This difference in height deficit between preterm infants in different social classes was not analysed using statistical tests. • There appears to be a stepwise increase in height deficit between ABW and LBW infants at ages 7, 11 and 23: preterm infants in social classes I & II have the lowest height deficit, followed by preterm infants in social classes IV & V, term infants in social classes I & II and finally term infants in social classes IV & V. • The height deficit in preterm infants in social classes I & II decreases during puberty between 7 and 16 years, whereas it increases for preterm infants in social classes IV & V.			
**Holmqvist** ^20^	Using Student’s t tests to compare the weekly growth of infants of well educated mothers with infants of less well educated mothers did not show significant differences for any of the time periods. 2. Between birth and 3 months, and 3–7 months, infants of well educated mothers had the highest mean weight increments of all the different infant categories: infants with well educated and less well educated mothers; infants with and without intrapartum foetal acidosis (scalp pH < 7.2); infants with and without neurodevelopmental disorders; infants with GA < 34 weeks and ≥ 34 weeks; male and female infants; all preterm infants in the cohort. • No formal statistical analysis (e.g. ANOVA) was used to compare multiple infant categories. 3. Between birth and 3 months, 3–7 months and 7–12 months, infants of well educated mothers had the highest mean length increments of all infant categories. • No formal statistical analysis (e.g. ANOVA) was used to compare multiple infant categories.			
**Ghods** ^21^	In a multivariable logistic regression analysis, Maternal education, the Home Facilities rating, and the Financial Situation rating, were significantly associated with head circumference (HC) catch-up (*p* = 0.012, *p* = 0.027, *p* = 0.001 respectively). Compared to infants without HC catch-up, those with HC catch-up were: • More likely to have mothers with education ≥ 12 years. • More likely to have a Home Facilities rating of Adequate and less likely to have a rating of Inadequate or Good. • More likely to have a Financial Situation rating of Good, and less likely to have a rating of Enough or Inadequate.			
**Ni** ^22^	While assessing the impact of differential loss to follow-up by socioeconomic status in the Discussion, the authors state, “Lower SES (socioeconomic status) was neither related to birth weight nor outcomes in our study but was associated with greater weight gain from birth to term age.” This association is not mentioned elsewhere and the relevant growth data are not reported. 2. Parental occupational SES moderated the association between birthweight SD score and BMI at 19 years: among participants with lower parental socioeconomic status, there was an inverse association between birthweight z-score and BMI at 19 years which remained significant after adjusting for sex, maternal age and maternal smoking during pregnancy. There was no such association in the higher socioeconomic status group. 3. Table S1 in the Supplementary Information shows the distribution of BMI at 19 years of age in infants with low and high parental occupational SES. Although there is no formal statistical comparison, those with parents of lower SES appear to have higher BMIs, and are particularly overrepresented among participants with BMI > 30.			
**Liang** ^25^	1. Analysis of variance showed a significant difference in the weight, length and head circumference of infants at 12 months according to average monthly household income (all *p* < 0.001). The reported growth measurements were as follows:			
	Monthly income:	< 3000 CNY	3000 – 5000 CNY	> 5000 CNY
	Weight (kg), mean ^36^	9.63 (0.964)	10.2 (0.924)	10.3 (1.39)
	Height ^65^, mean ^36^	71.9 (3.0)	73.7 (2.8)	73.7 (3.7)
	Head circumference ^65^, mean ^36^	44.2 (1.3)	44.9 (1.2)	0.9 (1.6)
	2. Multiple stepwise regression analyses showed that average monthly household income was significantly associated with weight (p = 0.001), body length (p = 0.029), and head circumference (p < 0.001) at 12 months after adjusting for other variables which were significant in univariate analyses, i.e. birthweight, feeding mode (breastfeeding, no breastfeeding or mixed feeding at 12 months) and sex for weight; intervention group (family integrated care vs usual care), gestational age, birth length and sex for body length; and intervention group, gestational age, birth HC and sex for HC.			
**Fu** ^24^	1. Chi squared analyses showed no significant association between maternal education level or maternal occupation and the presence of overweight/obesity at 4 to 7 years corrected age.			
**Sices** ^26^	1. Chi squared analyses showed no significant association between maternal education level and the occurrence of growth failure during any of the three study periods (40 weeks to 4 months, 4 to 8 months, 8 to 20 months).			
**Peterson** ^27^	1. Chi squared analysis showed no significant association between maternal education and the prevalence of subnormal head circumference at school age (mean age 6.8 years corrected age +/− 0.9 years).			
**Kelleher** ^23^	1. Chi squared analysis showed a significant association (*p* < 0.05) between maternal education and the incidence of failure to thrive (FTT) up to 36 months corrected age. Mothers of infants with FTT were less likely to have some college education but not a college degree, and more likely to have a college degree or postgraduate education than mothers of infants without FTT. 2. Chi squared analysis showed no significant association between family income and the incidence of FTT in the cohort. 3. A multivariable logistic regression analysis showed that maternal education ≥ college graduation compared some college education without a college degree was significantly associated with an increased risk of the infant developing FTT (*p* < 0.005) after adjusting for small for gestational age at birth, abnormal or suspect neurologic exam at birth, birthweight, maternal age, maternal height, and whether or not the infant lived with their father.			

Ahn et al. showed that the weight gain of infants born to employed mothers was slower than that of infants born to unemployed mothers from birth to 6 months (*p* < 0.001). Bocca-Tjeertes et al. showed that a low level of maternal education, but not low family income, was associated with restricted head circumference growth at 1 year compared to the population average. Teranishi et al. showed that at all time points (7, 11, 16 and 23 years), the height deficit between low birthweight (LBW, < 2500 g) and appropriate birthweight (ABW, ≥ 2500 g) preterm infants in the higher social classes was lower when compared with the height deficit between LBW and ABW preterm infants in the lower social classes.

Ghods et al. demonstrated that HC catch-up was significantly positively associated with a longer duration of maternal education (*p* = 0.012), and also with the families’ Home Facilities (*p* = 0.027) and Financial ratings (*p* = 0.001). Liang et al. showed that infants from households with the lowest monthly income ( < 3000 CNY) had the lowest weight, body length and head circumference measurements at 12 months.

Ni et al. reported that parental occupational SES was a moderator of the relationship between birthweight and BMI at 19 years of age in preterm-born participants: an inverse association was observed between birthweight z-score and BMI at 19 years in participants with lower SES and no association was observed among participants with higher SES.

Kelleher et al. reported that maternal education, but not annual family income, was associated with the incidence of failure to thrive (FTT) up to 36 months corrected age. Interestingly, the multivariable regression showed that infants of mothers with a college degree or higher education were at increased risk of FTT compared to infants of mothers with college education without a degree (reference group), after adjusting for demographic and birth variables.

Five studies did not show an association between parental SES and the postnatal growth of preterm infants. Sammy et al. demonstrated no statistically significant difference in the odds of growth deficit at 2 weeks post-discharge from the newborn unit between infants born to mothers with primary, secondary and tertiary level education compared to mothers with no education. Holmqvist et al. showed no significant differences in the weekly growth increments in weight, length or head circumference of infants of well educated mothers compared with infants of less well educated mothers. Fu et al. Sices et al. and Peterson et al. showed that there was no significant association between maternal educational level and the prevalence of overweight/obesity at 4 to 7 years corrected age; the incidence of growth failure up to 20 months; and the prevalence of subnormal head circumference at school age respectively.

### Risk of bias

The risk of bias within studies is summarised in Table 3.

**Table 3:** Assessment of bias in studies.

	Selection bias	Confounding bias	Missing data, attrition bias	Detection bias
**Ahn** ^16^ (methodology published in Ahn ^101^(Ahn, #93))	Excluded “socially vulnerable” premature infants, including those born to unmarried mother ^101^.	Infants of unemployed mothers were significantly heavier and longer at birth than infants of employed mothers (179 g and 1.1 cm difference between means). Birthweight and birth length were not adjusted for when analysing the effect of maternal employment status on growth variation. Other variables also shown to be associated with growth variation (e.g. problem at birth, problems at discharge and sex) were also not adjusted for in this analysis.	The number of infants for whom anthropometric measurements were taken at 6 months corrected age is not reported. When direct measurement was not possible at any time point, corresponding growth data were collected from the records.	Weight, height and head circumference measurements were taken at the same time of day according to standard guidelines. Measurements were taken twice to ensure consistency.
**Risk of bias (Ahn)** ^16^:	High	High	Unknown	Low
**Bocca-Tjeertes** ^17^	• Children were recruited from 13 community healthcare centres, covering approximately 25% of all children in the Netherlands, at the 4 year well-child visit attended by 97% of children. • Mothers in the non-response group were more likely to be of non-Dutch ethnicity and have a lower level of education.	Differences in baseline characteristics between infants of mothers with different levels of education, or infants in families with different levels of income, were not reported. Maternal height and small for gestational age status were significantly associated with head circumference growth restraint at 1 year, but not adjusted for in the multivariable logistic regression analysis of the association between maternal education and head circumference growth restraint.	20% of measured growth data was missing for children at each time point.	The fourth Dutch growth survey was used as a comparator group to determine growth restraint. Children with birthweight less than 2500 g, those with non-Dutch parents, those with diagnosed growth disorders or on medications known to affect growth were excluded in this survey ^102^.
**Risk of bias (Bocca-Tjeertes)** ^17^	High	Moderate	High	Low-Moderate
**Sammy** ^18^	All infants discharged from the newborn unit between April and June 2014 appear to have been screened. Only 3 out of 115 infants screened were not enrolled in the study.	Differences in baseline characteristics between infants of mothers with different levels of education were not reported. Gestational age, shown to be associated with the early growth of preterm infants, is not adjusted for in the analysis of the association between maternal education level and growth.	6 of 112 infants (5%) did not have their growth assessed at 2 weeks post discharge from the neonatal unit – there were 2 neonatal deaths, and 4 infants were lost to follow-up.	Infants were assessed at 2 weeks post discharge from the neonatal unit for the presence or absence of growth restraint. The timing of follow-up was not standardised. The outcomes “optimal growth” and “growth deficit” are not defined.
**Risk of bias (Sammy)** ^18^	Low	High	Low	High
**Teranishi** ^19^	Data from nationwide a cohort study of white, singleton children born in 1958 with perinatal and 23 year data available were used.	Differences in baseline characteristics between preterm infants in social classes I & II, and IV and V, were not reported. The associations of other variables with height deficit – the growth outcome reported by parental socioeconomic status – are not investigated.	Height data were available for: • 83% of subjects at 7 years. • 84% of subjects at 11 years. • 75% of subjects at 16 years. • 95% of subjects at 23 years. • The number and characteristics of preterm-born subjects for whom height data were available at each time point is unknown.	Heights were measured at ages 7, 11 and 16. Heights were self-reported at age 23.
**Risk of bias (Teranishi)** ^19^	Moderate	Unknown	Unknown	High (due to self-reported height at age 23).
**Holmqvist** ^20^	Only “low risk” infants without any maternal or foetal complications were included. The characteristics of infants lost to follow-up from the original cohort of 61 preterm infants^103^ are not reported.	Infants of well-educated mothers had a higher gestational age than those of less well-educated mothers (34 versus 33 weeks); no statistical comparison is reported. No confounding factors were adjusted for in analyses of the association between maternal education level and growth outcomes, but the presence of a neurodevelopmental disorder was associated with weight growth increments from 0 – 3 months and 3 – 7 months.	The number of infants for whom growth data was available at each time point, and their characteristics, are not reported.	The use of chronological versus corrected age to determine timing of follow-up is not specified. Age ranges of infants at follow-up appointments are not reported. Student’s t tests were used to compare weekly growth within discrete time periods instead of statistical methods which allow comparison of growth trends over the entire study period.
**Risk of bias (Holmqvist** ^20^**)**	High	High	Unknown	High
**Ghods** ^21^	65 of 238 eligible infants (27%) were excluded due to loss to follow-up (11%), incomplete medical records (14%), and inability to intrapolate the head circumference at 1 year (3%). It is unclear whether infants excluded due to loss to follow-up were lost before 1 year or up to 66 months corrected age.	Differences in baseline characteristics between infants in different socioeconomic strata were not reported. Other factors associated with head circumference (HC) catch up (e.g. birthweight z-score, birth length z-score, birth HC z-score, gestational age, small for gestational age status, the number of hospital days, neonatal comorbidities, breastfeeding characteristics) were not adjusted for in the analysis of the association between maternal education and HC catch up.	HC measurements for 9 infants (5%) were interpolated from adjoining data. The characteristics of these infants are not reported.	The presence of HC catch up is determined by the measurement at 1 year corrected age, but the age range of infants at the 1 year follow-up is not reported. Figure 1 of the study shows that infants’ head circumference z-score varies notably over time. The method used to determine families’ Home Facilities and Financial Situation was interviewer-dependent. The language used during the psychologist interview to measure parental socioeconomic status is not specified; over 40% of infants were from immigrant families.
**Risk of bias (Ghods** ^21^**)**	Moderate	High	Low	High
**Ni** ^22^	The cohort was recruited from a birth cohort study, and participants without data at 19 years (the last follow-up) were excluded. Participants with data at 19 years were more likely to have mothers with higher education and parents with higher occupational socioeconomic status compared to the original cohort.	Differences in baseline characteristics between infants of mothers with different educational attainment, or between infants of parents with different occupational socioeconomic status were not reported. Change in weight z-score from 2.5 to 6 years, shown in a regression analysis to be significantly associated with BMI at 19 years, was not adjusted for when reporting BMI by parental socioeconomic status.	There was no missing growth data at 19 years as this was specified as an exclusion criterion.	Only BMI, a calculated product of weight and height, is presented according to parental socioeconomic status. No other growth outcomes are reported.
**Risk of bias (Ni** ^22^**)**	High	High	Low	High
**Liang** ^25^	Inclusion criteria detailed that parents should have had “basic reading and comprehension skills”. The exclusion criteria excluded infants with severe illness, e.g. those with major comorbidities, infants who received invasive respiratory therapy, and infants who required surgery.	Differences in baseline characteristics according to primary caregiver education, and monthly household income, were not reported. No other variables shown to be associated with growth at 12 months were adjusted for when growth outcomes were reported according to monthly household income. The multiple regression analyses of growth outcomes at 12 months included other covariates significant in univariate analyses in addition to monthly household income. Ambiguity relating to covariates included in regression analyses (e.g. lack of reference time point for “feeding mode” variable) entails a risk of residual confounding.	Table 2 in the study appears to indicate that all 115 infants in the intervention group, and all 100 infants in the control group were followed-up at all time points until 18 months of age. However, it is not specified whether lack of attendance at a follow-up visit was a criterion for exclusion. The authors report a “20% loss of follow-up rate” in the Methods – it is unclear whether this was an estimated or actual rate.	The time range for which measurements were taken at the 12 month time point is not reported. It is not specified whether the time points refer to corrected or postnatal ages.
**Risk of bias (Liang** ^25^**)**	High	High for raw growth outcomes reported according to monthly household income. Moderate for multiple regression analyses.	Unknown	Low-Moderate
**Fu** ^24^	The characteristics of those excluded due to lack of data for assessment of childhood overweight/obesity, or missing data on study variables, is not reported. As the sample were recruited from cohort enrolled at birth, lack of attendance at the 4 – 7 year follow-up visit is likely to be associated with greater socioeconomic deprivation ^35,36^.	Differences in baseline characteristics according to maternal occupation and maternal education status are not reported. The categorisation of the maternal occupation variable (which includes the category “Others”) entails a risk of residual confounding. Other variables shown to be significantly associated with childhood overweight/obesity in univariate analyses (e.g. birthweight, birth length, formula-feeding, trajectory of BMI z-score during the first year of corrected age) were not adjusted for when reporting the incidence of childhood overweight/obesity by parental socioeconomic status.	There was no attrition as lack of data for the detection of childhood overweight/obesity at 4–7 years was specified as an exclusion criterion.	No other growth outcomes apart from childhood overweight/obesity are reported according to parental socioeconomic status.
**Risk of bias (Fu** ^24^**)**	Moderate	High	Low	High
**Sices** ^26^	The inclusion and exclusion criteria are reasonable to allow for the measurement of the specified growth outcomes, however, infants without at least two out of three consecutive growth measurements were excluded. The characteristics of the excluded infants are not reported.	Differences in baseline characteristics according to maternal education are not reported. Other variables shown to be associated with growth failure (e.g. chronic lung disease, cerebral palsy) are not accounted for when reporting the prevalence of growth failure during the different time periods by maternal education level.	All infants were assessed at 40 weeks, 93% of infants were seen at 4 months, 90% were seen at 8 months and 98% were seen at 20 months. 82% of infants attended all 3 visits after 40 weeks. The characteristics of those missing data at follow-ups is not reported.	Growth outcomes relating to length and head circumference are not reported. The comparisons between infants with growth failure in any study period and no growth failure is not reported. For the 5 infants (3%) with growth failure in more than one time period, only the first instance of growth failure is considered in the analysis; the parental socioeconomic status of these infants is not reported.
**Risk of bias (Sices** ^26^**)**	Moderate	High	Low-Moderate	Low-Moderate
**Peterson** ^27^	Loss to follow-up in the prospectively recruited cohort of very low birthweight infants, and the characteristics of those lost to follow-up, is not reported. As over 60% of the initial sample were recruited from the cohort enrolled at birth, lack of attendance at the school-age follow-up visit is likely to be associated with greater socioeconomic deprivation ^35,36^.	Differences in baseline characteristics according to maternal education status are not reported. Other variables shown to be associated with subnormal head circumference (HC) at school age (e.g. birthweight, small for gestational age status, neonatal risk score, cerebral palsy and overall neurosensory impairment at school age) were not adjusted for when reporting the incidence of subnormal HC by parental socioeconomic status. Authors report provided “health data” at the school-age assessment, but only report the rates of cerebral palsy and overall neurosensory impairment between children with and without subnormal HC – data for other comorbidities are not reported.	All children had school-age head circumference measurements as lack of this measurement was specified as an exclusion criterion.	Growth outcomes for weight and height are not reported by parental socioeconomic status; 37% of infants with subnormal HC also had subnormal weight and height (less than 2 SD below the mean). The school-age measurements took place between 5.9 and 9 years, but the analysis of subnormal HC by parental socioeconomic status does not account for the time at which the measurements were taken. Blinding of assessors to infant birthweight status (particularly as authors hypothesised that those with birthweight < 750 g would be more likely to have subnormal HC) when measuring HC is not specified.
**Risk of bias (Peterson** ^27^**)**	Moderate	High	Low	High
**Kelleher** ^23^	Infants were excluded if they were lost to follow-up before 30 months, if their mothers could not communicate adequately in English, or if their mothers reported drug or alcohol abuse or psychiatric hospitalisation. These criteria disproportionately exclude participants who are socioeconomically deprived^33–36^. Infants requiring intensive medical intervention (e.g. hospitalisation > 60 days after 40 weeks corrected age, or oxygen support for > 90 days) and those with severe neurodevelopmental abnormalities were also excluded.	Differences in baseline characteristics according to maternal education status or family income are not reported. The association between maternal education and the incidence of failure to thrive (FTT) is not adjusted for comorbidities even though children with FTT appear to be overrepresented among those with cerebral palsy, bronchopulmonary dysplasia and congenital heart disease.	The percentages of infants who attended each follow-up visit for growth outcome assessment, and the characteristics of these infants, are not reported.	The blinding of assessors to the intervention group in the IHDP trial is not specified. The stepwise variable selection process used for the multiple logistic regression analysis may have failed to highlight family income as a significant independent variable due to collinearity with maternal education. The choice of reference group for the maternal education variable (some college education without a college degree) in the multivariable regression model does not allow for comparison of infants at the two extremes of maternal education ( < High school versus ≥ college education).
**Risk of bias (Kelleher** ^23^**)**	High	High	Unknown	Moderate

The reported associations (or lack thereof) between parental SES and growth outcomes in all twelve studies have a high risk of confounding bias. Ten studies did not report differences in baseline infant and parental characteristics for groups with differing parental SES; of the studies that did, neither adjusted for the detected differences in their analyses.

In their analysis of growth outcomes according to parental SES, only three studies^17,23,25^adjusted for birth characteristics and/or comorbidities. Nine studies^16,18–22,24,26,27^did not adjust for the variables shown by their own analyses to be significantly associated with the growth outcome(s) under consideration. The two studies which reported BMI or overweight/obesity as a growth outcome measured but did not adjust for postnatal growth trajectories in their analysis of the association between parental SES and BMI or overweight/obesity^22,24^.

Six of the twelve studies reported missing data^16–19,21,26^. The numbers of infants for whom growth data was available at each time point are not reported by Kelleher et al. or Holmqvist et al., but any missing data will have had a large impact on the analysis in the latter study due to the small sample size.

The risk of detection bias is significant in eight studies. The height at 23 years in Teranishi et al’s study was self-reported – self-reported height has been shown to overestimate actual height^29^. The primary growth outcomes reported in Sammy et al.’s study, “optimal growth” and “growth deficit”, were undefined. Furthermore, as the timing of follow-up in this study was not standardised, infants could have been at different points in their growth trajectories at follow-up – previous studies have demonstrated that the growth trajectories of preterm infants vary during the first few days to weeks of life^6,10–12^. Holmvist et al. used Student’s t-tests to compare weekly growth between infants of well-educated and less well-educated mothers within discrete time periods; they did not use statistical methods which allow comparison of growth trends over the entire study period (birth to 48 months) whilst accounting for correlation between repeated measures. The studies by Sammy et al. and by Holmqvist et al. are likely to be underpowered due to the small samples of infants born to mothers with no education (n = 7) and infants born to well-educated mothers (*n* = 8) respectively. Ghods et al. used two interviewer-dependent measures of parental SES in their analyses; it is not specified whether the interviews were administered in German or a language of the parents’ choice, despite 40% of infants being from immigrant families.

In the studies by Ni et al. and Fu et al. BMI – or childhood overweight/obesity as defined by BMI z-scores – were the only growth outcomes reported according to parental SES. The use of BMI for the measurement of body fat mass has been widely criticised^30^. Furthermore, the exclusive reporting of BMI did not offer any information about height or weight growth.

The risk of selection bias is significant in eleven studies. We considered all studies which were likely to disproportionately exclude socioeconomically minoritised participants to be at moderate or high risk of selection bias, even if the study design did not specify selecting participants based on parental SES. We discuss this in detail in the next section.

### Health equity impact assessment

Given the subject matter of this review, we considered the health equity impact of the included studies using the framework developed by Castillo and Harris^31–34^, although this was not specified in our registered protocol (Table 4). Prompted by the disproportionate impact of the COVID-19 pandemic on minoritised communities, this framework was developed by researchers with expertise in health inequities and community-partnered research to facilitate consideration of studies’ health equity impact during peer review. The focus areas in the impact assessment are based on published guidance on conducting and evaluating health inequity and community-based participatory research.

**Table 4:** Health equity impact assessment. SES: Socioeconomic status. FTT: Failure to thrive. HC: Head circumference.

	Community engagement and research partnerships	Recruitment, representativeness and generalisability	Contextualisation and interpretation of data
**Ahn** ^16^	None reported.	Exclusion of “socially vulnerable” premature infants. Inclusion of infants born at an urban University hospital, and exclusion of infants transferred from medical centres. Characteristics of infants lost to follow-up at 6 months (if any) are not reported.	Context provided regarding lack of official pregnancy leave in Korea, sociocultural factors which place childcare burden on mothers, and the 2011 OECD report highlighting that most Korean women have low quality jobs with the greatest income inequality by sex in any OECD nation.
***Impact (Ahn)*** ^16^	***Neutral****.* *No explicit efforts have been specified to engage with communities regarding participation in this study, as the sample was recruited from inborn preterm infants at a University Hospital and underwent a 6-month follow-up*.	***Negative****.* *Those experiencing socioeconomic minoritisation, and those from rural areas with lower SES* ^38^*would have been disproportionately excluded*.	***Positive****.* *The study allows interpretation of the adverse effect of maternal employment in the context of structural barriers placed on employed Korean mothers, rather than considering “employment” a proxy for socioeconomic security*.
**Bocca-Tjeertes** ^17^	Not reported, despite the study cohort being a community-based sample.	Recruitment from community healthcare centres covering 25% of all children in the Netherlands at a routine visit attended by 97% of children. However, mothers in the questionnaire non-response group more likely to be of non-Dutch ethnicity and have a lower education level.	Authors mention that low maternal education may be associated with low maternal HC, thereby suggesting a genetic mechanism for HC growth restraint for.
***Impact (Bocca-Tjeertes)*** ^17^	***Likely negative****. Building community partnerships for engagement may have increased the questionnaire response rate of socioeconomically minoritised people, including those of non-Dutch origin*.	***Negative***. *Data was disproportionately missing from those of non-Dutch ethnicities, known to also be socioeconomically minoritised*^78^, *and mothers with a lower education level. This may have underestimated the effect of socioeconomic minoritisation on growth restraint*.	***Negative****.* *The study subscribes to biological essentialism by not offering any non-biological explanations for the relationship between lower education level and HC growth restraint have been offered. Those experiencing socioeconomic and ethnic minoritisation have been suggested to have restricted access to specialised healthcare services in the Netherlands* ^78^.
**Sammy** ^18^	Not reported.	The sample was recruited at discharge from a District Hospital newborn unit serving a rural population in Kenya. 3 of 115 infants screened for inclusion were excluded due to congenital anomaly and lack of guardian consent. 4 of 112 infants were lost to follow-up, and there were 2 neonatal deaths.	Authors state that “education always empowers the mothers in all aspects and should therefore be emphasised”. Specific mechanisms by which educational empowerment may contribute to better health outcomes, and structural barriers to education for mothers are not discussed.
***Impact (Sammy)*** ^18^	***Neutral****.* *Building community partnerships for engagement may have facilitated attendance at follow-up for the 4 infants lost to follow-up, although these were a small proportion of the total sample (3.5%)*.	***Positive****.* *This study focused on preterm infants in rural Kenya, where a lower proportion of households are in the wealthiest national quintile compared to urban areas*^40^. *The exclusion criteria are not specifically associated with socioeconomic minoritisation. Although loss to follow-up* ^104^*and neonatal death* ^105^*are associated with socioeconomic minoritisation in Kenya, only 5% of infants were lost to follow-up in total*.	***Negative****.* *The study contributes to a deficit-based narrative of mothers with a low education level. In identifying the mechanisms by which health outcomes may be worsened for those experiencing minoritisation due to their education level, nor acknowledging structural barriers to education in rural Kenya, the study misses an opportunity to identify specific targets for intervention*.
**Teranishi** ^19^	Not reported.	UK birth cohort of white, singleton children born in 1958 was studied.	Brief mention of social determinants of health, e.g. education, nutrition, healthcare, which may have contributed to greater catch-up growth among preterm infants in the upper social classes. Authors acknowledge that “improved social conditions during gestational, infant and childhood periods” at the end of the Discussion.
***Impact (Teranishi*** ^19^***)***	***Likely negative****.* *No efforts to engage with communities regarding research participation and attendance at follow-up time points are specified. Given the long follow-up duration of 23 years, lack of community engagement is likely to have resulted in non-random attrition among socioeconomically minoritised participants* ^37^.	***Negative****.* *In addition to the likely exacerbated attrition of socioeconomically minoritised participants over the 23-year follow-up period, the exclusion of children from non-white race/ethnicities would have disproportionately excluded participants from disinvested communities. Poverty is known to be associated with race/ethnicity in the UK* ^106^.	***Neutral****.* *The study identifies social determinants of health as being targets for intervention throughout the lifecourse. However, despite the fact that social class was studied as the exposure in this study, the authors document health inequity and do not expand on how “social conditions” could be improved*.
**Holmqvist** ^20^	Not reported.	Exclusion of infants with maternal and foetal complications. Follow-up was based at an urban University hospital.	Authors state there may be “possible socioeconomic influences” contributing to the favourable growth of infants of well educated mothers from birth to 7 months.
***Impact (Holmqvist*** ^20^***)***	***Likely negative****.* *No efforts have been specified to engage with communities regarding participation in this study. Lack of community engagement may have affected differential attrition by SES over the 48-month follow-up*^35^, *especially as the follow-up visits required in-person attendance*	***Negative****.* *Maternal and foetal complications are both associated with socioeconomic minoritisation in Sweden*^103,107^, *resulting in disproportionate exclusion of socioeconomically minoritised participants. The study’s urban setting may have impacted the follow-up attendance of infants from rural communities*.	***Neutral****.* *Despite acknowledging “socioeconomic influences”, this study does not discuss the mechanistic pathways by which named influences may have affected the postnatal growth of preterm infants, thereby missing an opportunity to identify targets for intervention*.
**Ghods** ^21^	Not reported.	Infants lost to follow-up before 66 months were excluded. Over 40% of infants were from immigrant families. Follow-up was based at a University hospital in Vienna.	No context provided for the criteria in the Home Facilities (light, ventilation, accommodation etc) and Finance (education level, occupation and social facilities) assessments to justify their use, nor for the scale used to determine parental SES (Good, Adequate, Inadequate). The language used in the interview is not specified. Mention previous literature which shows an association between “family socioeconomic situation” and HC growth. Breastfeeding is suggested as a potential mediator for the relationship between higher maternal education and catch-up growth, but breastfeeding was not associated with maternal education in this study.
***Impact (Ghods*** ^21^***)***	***Likely negative****.* *No efforts to engage community members in research participation have been discussed; such efforts may have reduced the 11% loss to follow-up*.	***Negative****.* *Exclusion of infants lost to follow-up would have disproportionately excluded socioeconomically minoritised people who are more impacted by loss to follow-up*^35,36^, *and may have disadvantaged those from rural areas. The proportion of infants from immigrant families reflects the proportion of the population with a “migrant background” in Vienna in* ^108^.	***Negative****.* *The use of a compound measure of parental SES with a non-validated scale may obscure the health equity impact of certain factors (e.g. occupation versus education versus “social facilities”)*. *Lack of use of appropriate interpretation services during interviews to determine parental SES will likely have resulted in disproportionate misclassification among those who do not speak the language used, given 40% of the sample were immigrants. Further consideration of the association between the Home Facilities and Financial Situation ratings and HC catch-up growth may have identified targets for intervention*.
**Ni** ^22^	Not reported.	Participants lost to follow-up before 19 years were excluded, and participants with data at 19 years were more likely to have mothers with higher education and parents with higher occupational SES. This study included children born across maternity units in the UK and Ireland (276 centres in total).	Authors posit that pre-pregnancy maternal weight may mediate the relationship between lower parental SES, birthweight and BMI at 19 years.
***Impact (Ni*** ^22^***)***	***Likely negative****.* *The authors do not specify measures taken to engage community members in this research. These measures may have helped to address research mistrust and encouraged more minoritised participants invited to participate in the assessment at 19 year* ^109^.	***Negative****.* *Participants were recruited from maternity units based in urban and rural areas. Loss to follow-up over the extended 19 year follow-up period disproportionately affected socioeconomically minoritised participants* ^35,36^.	***Negative****.* *Although authors identify pre-pregnancy maternal weight as a potential mediator, they do not acknowledge the structural determinants of health which underlie the association between low parental SES and pre-pregnancy weight. An individualist explanation is offered without identifying wider determinants amenable to policy and/or social change*.
**Liang** ^25^	Not reported.	Only infants of parents with “basic reading and comprehension skills” were included. Infants with severe illness were excluded. Follow-up was based at a teaching hospital.	No context or explanation offered for the relationship between household income and growth outcomes at 12 months.
***Impact (Liang*** ^25^***)***	***Likely negative****.* *Given that the authors aim to study the feasibility and impact of family integrated care, community partnerships may have facilitated better informed consent from parents, particularly those from minoritised communities, for study participation*.	***Negative****.* *The study excludes infants of parents who experience minoritisation due to their education level, for whom family integrated care may have been particularly beneficial due to the paucity of accessible information and training on caring for preterm infants. Excluded infants with severe illness are more likely to be from socioeconomically minoritised communities*^110^. *Those from rural communities, who are more likely to have a lower SES*^38^, *may have faced additional barriers to follow-up attendance*.	***Negative****.* *Although this study was focused on family integrated care as the intervention, the authors miss an opportunity to discuss why monthly household income is significantly associated with all three measured growth outcomes (HC, weight and body length), and identify targets for intervention*.
**Fu** ^24^	Not reported.	Infants without data at the 4-7 year follow-up visit due to lack of attendance or loss to follow-up within the birth cohort were excluded.	No context offered regarding the findings on the lack of relationship between maternal occupation or education and overweight/obesity at 4-7 years.
***Impact (Fu*** ^24^***)***	***Likely negative****.* *Building community partnerships may have decreased the number of children for whom “data essential for defining childhood overweight/obesity” were unavailable at 4-7 years in this birth cohort study (4823 of 8269 eligible infants, 58%)*.	***Negative****.* *Socioeconomically minoritised infants would have been overrepresented among those who did not attend the 4-7 year visit or were lost to follow-up* ^35–37^.	***Negative****.* *The authors adjust for maternal education and occupation when examining the association between modifiable feeding practices and BMI trajectories, suggesting they consider these to be important confounders of this association. However, they do not comment on why maternal education or occupation was not associated with overweight/obesity at 4-7 years – it may be that these variables were not granular enough or contextually appropriate to capture the impact of SES in this study, and consequently there may be residual confounding due to socioeconomic minoritisation in the analyses*.
**Sices** ^26^	Not reported.	Infants without at least two consecutive growth measurements were excluded. 18% of infants who were discharged from the NICU did not attend all 3 follow-up visits. Follow-up was based at an urban teaching hospital.	No context offered for the lack of association between maternal education and growth failure during the three study periods. Maternal education is presented as part of a composite “social risk” score along with “African=American/Black” ethnicity and “unmarried” maternal status.
***Impact (Sices*** ^26^***)***	***Likely negative****.* *Community engagement in research participation may have facilitated greater attendance at follow-up visits up to 20 months’ corrected age*.	***Negative****.* *Loss to follow-up or lack of attendance would have occurred disproportionately among socioeconomically minoritised infants*^35,36^. *Infants from rural areas*^39^, *may have faced additional barriers to follow-up attendance*.	***Negative****.* *The authors do not explore mechanisms by which “social risk”, composed of non-modifiable factors, may contribute to postnatal growth failure, thereby failing to identify strategies for intervention. Having less than a high school education is positioned as being equivalently risky to “African-American/Black” ethnicity and “unmarried” status, ignoring the pathways of structural oppression and minoritisation associated with these individual factors, and the importance of intersection between these factors*.
**Peterson** ^27^	Not reported.	Infants with an unreliable or missing HC measurement at school age were excluded. Follow-up was based at an urban teaching hospital and two other tertiary centres.	No context regarding lack of association between maternal education and subnormal HC at school age. Maternal education less than high school, “non-white” ethnicity and “unmarried” marital status constitute a composite “sociodemographic risk” score.
***Impact (Peterson*** ^27^***)***	***Likely negative****.* *Building relationships with communities may have facilitated greater participation from minoritised communities at the school-age visit*.	***Negative****.* *Socioeconomically minoritised infants would have been more likely to have not attended the school-age follow-up visit*^35–37^. *Unreliable or missing HC measurements may have been more likely in infants from rural communities* ^39^.	***Negative****.* *Similar to the study by Sices et al, the authors do not discuss how “sociodemographic risk” may result in subnormal postnatal HC growth. The equivalation of unmarried status, “non-white” ethnicity and less than high school education again misses the tangible mechanisms by which structural oppressions operate and intersect*.
**Kelleher** ^23^	Not reported.	Exclusion of infants lost to follow-up before 30 months, infants with mothers who could not adequately communicate in English, infants with mothers reporting drug or alcohol abuse or psychiatric hospitalisation, infants requiring intensive medical intervention or severe neurodevelopmental abnormalities.	No context provided to explain the bimodal distribution of maternal education between the FTT and non-FTT infants, nor for the finding that advanced maternal education was associated with the development of FTT. No explanation offered for the lack of association between family income and FTT.
***Impact (Kelleher*** ^23^***)***	***Likely negative****.* *Building community relationships may have supported more minoritised participants to attend follow-up until 30 months*,	***Negative****.* *This disproportionately excludes socioeconomically minoritised participants* ^33–36^.	***Negative****.* *The authors do not account for their significant selection bias in explaining the association of advanced maternal education and FTT. They also do not discuss factors which may have led to disparate findings for the two indicators of SES, namely family income and maternal education. There is no discussion of contextual factors which may lead to one measure of SES being more suitable than the other, nor the different mechanisms by which maternal education level and family income may act to influence postnatal growth*.

Socioeconomically minoritised infants were underrepresented in eleven studies. Bocca-Tjeertes et al reported that the mothers in the non-response group were more likely to have a lower level of education. Several studies specified eligibility criteria which disproportionately excluded socioeconomically minoritised participants^16,20,23,25^. Finally, the studies which excluded infants lost to follow-up^19,21^ or infants without growth measurements at specific time points^22,24,26,27^ disadvantaged participants from disinvested communities^35–37^.

We also considered the impact of the study’s setting on representativeness. Ahn et al actively excluded infants born outside an urban University hospital, and Sices et al, Ghods et al, Holmqvist et al, Peterson et al and Liang et al conducted their follow-up at urban tertiary or teaching hospitals. This would have disproportionately affected the inclusion and follow-up attendance of participants from rural communities^38,39^. Conversely, the study by Sammy et al had a positive health equity impact as it was based in a District Hospital serving infants in rural Kenya^40^.

Only one study, by Ahn et al., had a positive health equity impact with regard to the contextualisation and interpretation of data. Both Sices et al. and Peterson et al used a composite “social” or “sociodemographic” risk score, but did not justify the use of these scores. Bocca-Tjeertes et al. invoked biological essentialism by proposing a genetic mechanism for the reported association between low maternal education and head circumference growth restraint. The studies by Sammy et al., Teranishi et al., Holmqvist et al. and Ghods et al. acknowledged the role of socioeconomic or sociocultural factors, but did not propose mechanistic pathways by which resultant minoritisation shapes exposure to structural determinants of health. Similarly, Ni et al. did not consider the structural determinants of pre-pregnancy weight that may mediate the relationship between lower parental socioeconomic status, birthweight, and BMI of preterm-born infants at 19 years.

Four studies^23,24,26,27^ did not offer an explanation for the reported lack of association between parental SES and growth outcomes. Whereas, Liang et al. and Ghods et al. did not discuss their findings of a relationship between parental SES and measured growth outcomes.

None of the studies reported methods used for community engagement in research participation.

## Discussion

This is the first systematic review to directly investigate the role of SES in the postnatal growth of preterm infants. Only twelve out of nearly 15,000 screened articles met the eligibility criteria as most studies did not explicitly report growth outcomes according to parental SES. The settings, growth outcomes, timings of growth outcome measurement, and measures of SES used in these studies were highly heterogeneous.

Six studies^16,17,19,21,22,25^reported a relationship between lower parental SES and patterns of growth in preterm infants likely to be associated with adverse longer term neurodevelopmental or metabolic outcomes, whereas five studies reported no association between parental SES and postnatal growth^18,20,24,26,27^. A single study, Kelleher et al., showed that infants of mothers with a college degree or greater education compared to mothers with some college education without a degree were at increased risk of developing failure to thrive^23^. However, the choice of reference group in the analysis (i.e. mothers with some college education, one of the intermediate categories) did not allow for a robust comparison of growth outcomes according to parental SES as those at the extremes of socioeconomic privilege and deprivation could not be compared.

All included articles have a high risk of bias. Ten studies do not report differences in baseline characteristics of infants born to parents of different SES categories, and only three studies adjust for birth characteristics or postnatal factors affecting the extrauterine growth of preterm infants, such as anthropometric measurements at birth^11^, and neonatal comorbidities including sepsis, chronic lung disease, postnatal steroid treatment, and necrotising enterocolitis^11,41,42^. Ni et al. and Fu et al. do not adjust for postnatal growth trajectories when analysing the relationship between parental SES and BMI at 19 years and 4 to 7 years respectively, even though there is evidence to suggest the rate of postnatal “catch up” growth may be associated with fat deposition^43^. Eleven studies suffer from selection bias as study participants are less likely to have a low SES.

There may also be a risk of residual confounding due to the choice of parental SES measure in the included studies. For example, the granularity of information regarding parental SES is often limited as several studies use binary classifications of maternal education^17,20–22,26,27^. Furthermore, although maternal education and parental occupation have been used widely as measures of SES, household income has been shown to specifically affect children’s cognitive, socio-behavioural and health outcomes^44^. Only three studies^17,23,25^ measure household income, and only two of these studies use a quantitative categorisation of income^23,25^. Notably, in the study by Liang et al., an association was seen between all three growth outcomes (height, weight and head circumference) at 12 months and monthly household income, whereas no association was seen with primary caregiver education. Kelleher et al. also use a three-level classification of family income as a measure of parental SES but do not demonstrate any association with infant growth, whereas there is an association between maternal education and infant growth. The association between income and growth may not have been revealed in the multiple regression analysis due to collinearity between the income and maternal education variables.

Poorer outcomes during neonatal intensive care unit (NICU) admission and a detrimental impact on parents and families may contribute to socioeconomic disparities in the postnatal growth of preterm infants. Despite the protocolised nature of neonatal intensive care, the adverse effects of socioeconomic deprivation are seen in the quality of care. In the United States, NICUs with a greater proportion of patients from a lower socioeconomic background, and units situated in more deprived areas, were more likely to have a lower Baby-MONITOR quality score^45^. Socioeconomic disparities have been noted in the outcomes of neonatal sepsis, a common diagnosis which often necessitates NICU admission^46^.

Differences in nutritional practices may also mediate inequities in the postnatal growth of preterm infants. Early nutrient delivery in the NICU has been shown to be associated with growth velocity in the neonatal period^47^. Breastmilk is known to reduce the risk of neonatal morbidities and improve neurodevelopmental outcomes in preterm infants^48–50^. However, socioeconomically minoritised mothers are less likely to initiate^51^ and continue breastfeeding preterm infants^52^.

The burden of unmet material needs among socioeconomically minoritised families is likely to contribute to adverse growth outcomes for preterm infants; in a cohort of low-income families with preterm infants across the US, 26% suffered from food insecurity, 33% from housing insecurity, and 28% from energy insecurity^53^. These material inequities result in obstacles to, and increase the opportunity cost of, practices such as breastfeeding and attending follow-up appointments.

Pathophysiological pathways may link the experience of stress in parents to poor growth outcomes in preterm-born children. Low SES has been shown to exacerbate the impact of negative life events on antenatal anxiety and depression^54^. Maternal antenatal stress exposure, in turn, was associated with white matter microstructural changes in preterm infants at term-equivalent age^55^.

Financial stress related to NICU care may also limit the involvement of parents from historically and contemporaneously excluded communities, and impact attendance at follow-up appointments^35^. In a prospective cohort study of 169 mother-infant dyads, an annual household income > $100,000 was associated with more time spent in the NICU^56^. Parents of preterm infants are at higher risk of reducing or stopping work^57^, particularly if their child has additional medical comorbidities^58^. Furthermore, parents of preterm infants face substantial travel and subsistence expenses whilst their infants are in the neonatal unit^59–61^.

Accordingly, strategies to reduce the duration of inpatient hospitalisation may be effective in alleviating some of the financial burden faced by parents. Breastfeeding rates could be maintained with adequate nursing support following early discharge of preterm infants from the NICU once full oral feeding had been established^62^. In preterm infants requiring tube feeding, conventional NICU inpatient care and home tele-healthcare resulted in comparable weight for age z-scores at discharge and exclusive breastfeeding rates^63^. On the other hand, unidimensional strategies tackling a single cost (e.g. parking charges) are unlikely to be effective^64^.

Individual and neighbourhood-level socioeconomic deprivation continue to affect the health of preterm infants throughout childhood. Ex-preterm children are at higher risk of hospitalisation for bronchiolitis^65^and poverty has been shown to be associated with poorer outcomes in critically ill children admitted with bronchiolitis^66^. Preterm-born children with bronchopulmonary dysplasia living in the most deprived areas in Philadelphia had increased Emergency Department visits, hospital readmissions and activity limitations compared with those in the least deprived areas^67^. Despite the use of regional follow-up protocols after preterm birth in a setting with well-developed healthcare infrastructure, very preterm-born children living in deprived neighbourhoods in France had more frequent unplanned rehospitalisations^68^.

Considering the high-intensity clinical support offered in the NICU, there is potential to identify and address individual and systemic barriers in a standardised manner to optimise outcomes for socioeconomically deprived patients. In 2016, the American Academy of Pediatrics recommended universal screening for adverse social determinants of health in paediatric services and onward referral if required^69^. However, Parker et al. ‘s mixed methods study in two NICUs in “safety-net” hospitals in the US published in 2021 showed that, apart from employment, other unmet basic needs including food insecurity, were not commonly assessed^53^. The implementation of a standardised screening tool integrating screening and referral into clinical workflow increased systematic screening from 0% to 49%, with 98% of families requesting assistance receiving referrals^70^. It has been shown that referrals following screening for basic unmet needs at community paediatric outpatient appointments leads to greater receipt of community resources for families^71^. They also advocate for non-siloed holistic care through screening for postpartum depression and broader screening for parental mental health needs in the NICU^72^.

The analysis in this review is limited by the fact that we did not examine outcomes by race/ethnicity in the included studies. When considering the communities affected most by socioeconomic minoritisation, it is crucial to consider the impact of structural racism, how it intersects with economic disinvestment, and how it disproportionately exposes adversely racialised people to poverty and its consequences. A well-known manifestation of structural racism is stress. As described by Arline Geronimus^73^, “weathering” during pregnancy – and throughout the lifecourse –may exacerbate stress-related physiological changes in foetuses^55^, and worsen the physical and mental health of parents facing combined socioeconomic and racial minoritisation. Furthermore, a systematic review highlighted that black and Hispanic infants in the US were more likely to be cared for in hospitals providing lower quality care with greater neonatal morbidity and mortality^74^. However, there were also notable within-NICU inequities. In two studies, Black and Hispanic infants were less likely to be referred for follow-up care than white infants. Racial/ethnic inequities were found in NICU breastfeeding rates, with limited breastfeeding education and support listed as contributory factors. Qualitative research with Black birthing people has shown through examples how structural, institutional and interpersonal racism affected the quality of NICU care^75^. A health system-focused strategy is required to mitigate racial/ethnic inequities in NICU care, including standardised assessments of structural determinants of health, psychosocial support, and support for material needs^76^.

The studies in this review had a largely negative impact on health equity. In addition to selection bias which disproportionately excluded socioeconomically minoritised participants, those from rural communities may have been impacted by the eligibility criteria and follow-up arrangements for studies based in urban centres^16,20,21,25–27^. This is likely to have differential effects based on local socioeconomic geography. In Korea and China, those in rural areas experience greater socioeconomic minoritisation^38^, and in the US, those from rural communities are less likely to attend paediatric follow-up appointments^39^. In western and Nordic European countries, the highest risk of poverty was recorded in people living in cities^77^. Conversely, Sammy et al.’s study is based in a District Hospital in rural Kenya, where fewer households are in the wealthiest quintile compared to urban areas^40^.

Theorisation concerning mechanisms by which socioeconomic minoritisation may be affecting postnatal growth is lacking throughout the studies. Only Ahn et al. offer context to explain why the policy and sociocultural environment in Korea systematically disadvantages infants of employed mothers. Opportunities were otherwise missed to identify the processes by which socioeconomic minoritisation actively shapes exposure to structural determinants of health, and to identify targets for intervention and policy change. The genetic link between head circumference growth restraint and low maternal education proposed by Bocca-Tjeertes et al. fails to consider modifiable structural factors; for instance, those experiencing racial/ethnic and socioeconomic minoritisation in the Netherlands have been shown to face barriers accessing specialised health services^78^. Furthermore, Sices et al. and Peterson et al. use a composite risk score, positioning the minoritisation from a low maternal education level as equivalent to racial/ethnic minoritisation and stigmatisation due to “unmarried” status. The use of these composite scores ignores the pathways by which different structural oppressions may manifest and intersect.

None of the studies report efforts to engage or co-produce research with communities, including the study by Bocca-Tjeertes et al. based in community health centres, or studies which have a very long follow-up period, such as the 23 year follow-up by Teranishi et al. Co-design with community stakeholders may have mitigated loss to follow-up seen disproportionately in socioeconomically minoritised participants^35–37^by allowing researchers to identify and address exclusionary research practices^79^.

Furthermore, the lived expertise of community members may highlight locally relevant mechanisms by which health inequity due to socioeconomic minoritisation is (re)produced and maintained. In fact, collaboration with community advocates as a means of redistributing power to minoritised communities has been highlighted as a key strategy to avoid harmful health equity “tourism”^80^. The mechanisms by which lower SES affects preterm infant growth are likely to vary depending on health system infrastructure, political context and intersecting oppressive structures. For example, the cost of accessing healthcare services^81^may result in catastrophic health expenditure and reduced spending on essential items including food^82^, thereby affecting infant growth through food insecurity^83^. Even if healthcare is offered free of cost, preterm birth is associated with the stress of significantly increased economic costs over the first 2 years^84^, which are likely to be exacerbated with rising costs of living. In India, minoritisation due to caste is associated with higher infant and child mortality rates^85^. In a study of mothers of preterm infants in Malaysia, Malay, Indian, Chinese and indigenous ethnicities had different positive and negative associations with quality of life in various domains, including physical health, psychological wellbeing and the quality of their environment^86^.

When co-designing future research, researchers should consider how to capture socioeconomic minoritisation as an exposure, to prevent deficit thinking that avoids consideration of the structural determinants of health^87^. Variables of interest may include exposure to economic policies (e.g. austerity) associated with greater material impoverishment^88^, racial-economic segregation resulting from structural racism^89^, and employment precarity, which is linked to adverse workplace health outcomes^90^. Given the significance of the intersection between socioeconomic minoritisation and adverse racialisation, future studies investigating the former should also consider measuring structural racism as an exposure^91^.

## Conclusion

The impact of parental SES on the postnatal growth of preterm infants may be a mechanism for the intergenerational transfer of health inequity in socioeconomically minoritised communities. In our systematic review, limited and low certainty evidence suggests that socioeconomic minoritisation may adversely affect the postnatal growth of preterm infants. However, the high risk of bias and lack of adjustment for confounding factors in the included studies compromises the reliability of their findings. Primary observational studies measuring socioeconomic minoritisation, other intersecting oppressions, and antenatal and neonatal factors which are known to impact postnatal growth, will help to identify further targets for intervention and policy change.

## Supplementary information

Supplementary information

## Data Availability

All data generated or analysed during this study are included in this published article [and its supplementary information files].
