# Supplementary material for: Socioeconomic disparities in the postnatal growth of preterm infants: a systematic review
Source: Pediatr Res. 2024 Jul 18;97(2):532–57. doi: 10.1038/s41390-024-03384-0 (PMC12014492; doi:10.1038/s41390-024-03384-0)
Supplement: Supplementary file 1 — Supplementary information [file 41390_2024_3384_MOESM1_ESM.pdf]

Supplementary Table 1: Inclusion and exclusion criteria

	**Inclusion criteria**	**Exclusion criteria**
Study design	Original research studies reporting data from humans, including clinical trials and observational studies.	Case report, commentary, editorial, comment, review (systematic or otherwise), report, guidelines, position paper<br>**OR**<br>Studies that do not report data relating to humans.<br>**OR**<br>Basic science studies without any patient data.
Population	Preterm infants born at less than 37 weeks gestational age at birth.	Infants born at greater than 37 weeks gestational age at birth, infants of unknown gestational age.
Exposure	Report of parental socioeconomic status through any means, including education, household income, address.	No report of parental socioeconomic status.<br>**OR**<br>Parental socioeconomic status only adjusted for in multivariable analyses, and therefore not explicitly reported.
Outcome	Growth of infants measured using anthropometric measures, e.g. length, weight, abdominal circumference, head circumference **AND** reported according to parental socioeconomic status.	Anthropometric growth outcomes not reported.<br>**OR**<br>Growth outcomes not evaluable according to parental socioeconomic status.

Supplementary Table 2: Search strategies used for the systematic review in the Medline, EMBASE, CINAHL Plus and Web of Science databases. For the Medline and EMBASE searches, free text terms and index-terms within each domain (population, exposure and outcome) were combined with "OR" and terms across domains were combined with "AND".

**Database**	**Population**	**Exposure**	**Outcome**	**Study design**
Medline, EMBASE – free text terms	Preterm<br>Pre-term<br>Prematur*<br>"Low birth weight"	Socioeconomic<br>Socio-economic<br>Poverty<br>Inequalit*<br>Inequit*<br>Disparit*<br>Income<br>Standard* adj2 living<br>Deprivation adj3 (index OR indices)	Growth<br>Weight gain<br>Weight<br>Length<br>Head circumference	"Case control"<br>Cohort adj (study or studies)<br>"Cohort analy$"<br>"Follow up" adj (study or studies)<br>Observational adj (study or studies)<br>Longitudinal<br>Retrospective<br>"Cross sectional"<br>Clinic$ adj trial$1<br>**NOT**<br>"Case report"
Medline, EMBASE – index terms	Infant, Low Birth Weight (exp)<br>Infant, Premature (exp)	Socioeconomic Factors (exp)<br>Social Marginalisation (exp)<br>Poverty areas (exp)<br>Healthcare Disparities (exp)	Growth (exp)<br>Body size (exp)<br>Weights and Measures (exp)<br>Anthropometry (exp)	Epidemiologic studies<br>Case control studies (exp)<br>Cohort studies (exp)<br>Cross-sectional studies<br>Clinical trial<br>Clinical trials as Topic (exp)<br>**NOT**<br>Letter OR<br>Historical article OR<br>Comment OR<br>Editorial OR<br>Expression of concern OR<br>News OR<br>Patient education handout OR<br>Retraction of publication OR<br>Case reports OR<br>Consensus development conference
Web of Science Core Collection	TS=(preterm OR "pre-term" OR "prematur*" OR "low birth weight")	TS=(Socioeconomic OR "Socio-economic" OR Poverty OR Inequalit* OR Inequit* OR Disparit* OR (Standard* NEAR/2 living) OR Income OR (Deprivation NEAR/3 (index OR indices)))	TS=(Growth OR weight OR "weight gain" OR length OR "head circumference")	TS=("Clinical trial*" OR (clinic* NEAR trial*) OR "case control" OR (cohort NEAR (study OR studies)) OR "cohort analy*" OR ("follow up" NEAR (study OR studies)) OR ("follow-up" NEAR (study OR studies)) OR (observational NEAR (study OR studies)) or longitudinal OR retrospective OR prospective OR "cross-sectional" OR "cross sectional")**NOT** TS=("case report")
CINAHL Complete	TX (preterm OR "pre-term" OR premature" OR "prematurity" OR "low birth weight")	TX (Socioeconomic OR "Socio-economic" OR Poverty OR Inequality OR Inequalities OR Inequity OR Inequities OR Disparity OR	TX (Growth OR "Weight gain" OR Weight OR Length OR "Head circumference")	PT ("Clinical trial" OR "Clinical trials" OR "Cohort study" OR "Cohort studies" OR "Case control" OR "Case-control" OR "Cross sectional" OR "Cross-sectional" OR "Follow up study" OR "Follow up studies" OR "Follow-up study" OR "Follow-up studies" OR "Cohort analysis" OR "Cohort analyses" OR

		Disparities OR "Living standard" OR "Living standards" OR "Standard of living" OR "Standards of living" OR Income OR "Index of deprivation" OR "Index of multiple deprivation" OR "Indices of deprivation" OR "Indices of multiple deprivation") **AND**		"Observational study" OR "Observational studies" OR Longitudinal OR Retrospective OR Prospective)<br>**NOT** PT ("Case report" OR "Case study" OR Editorial OR Comment OR Commentary OR "Retracted publication")

Supplementary Table 3: Data collection form used to extract data from studies.

**Reference and Design**	**Population**	**Exposure**	**Outcome measures**
Author:	Inclusion criteria:	Measure of parental socioeconomic status:	Primary outcomes, and time point(s) at which measured:
Publication year:	Exclusion criteria:		
Country:	Number of preterm infants < 37+0 gestational age:	Methods for measuring/deducing parental socioeconomic status:	Secondary outcomes, and time point(s) at which measured:
Study question:	Calculation of GA:		
Study design:	Other non-preterm participant groups and numbers:	Is parental socioeconomic status explicitly reported for preterm infants?	Anthropometric growth outcomes measured for preterm infants, and time point(s) at which measured:
Number of study centres:			
Setting of centres (e.g. urban, rural, mixed, unknown):	Gestational age of preterm infants at birth:	If parental socioeconomic status not explicitly reported, is it used in the data analysis elsewhere?	Methods for assessing anthropometric growth outcomes in preterm infants:
Funding:			Are growth outcomes reported separately by parental socioeconomic status?

Supplementary Table 4: Tool used to assess the risk of bias in included studies.

Did the study address a clearly focused issue?	
Was the cohort recruited in an acceptable way?<br>- Inclusion/exclusion criteria	
If there was a comparison group, was it appropriate?<br>- Does the strategy for recruiting participants differ across groups?<br>- Were inclusion/exclusion criteria the same across groups?	
Was the socioeconomic status measured using a valid and reliable measure?	
Were the growth outcomes measured using valid and reliable measures?<br>- Were they assessed blind if comparison group present?	
Have all important confounding factors been measured?	
Have they taken account of the confounding factors in the analysis?	
Was the follow up of subjects complete enough?<br>- Differences in characteristics between those retained vs those lost to follow up/those for whom outcomes not available at follow up<br>- With differential losses to follow up, was the impact assessed (e.g. sensitivity analysis/adjustment methods)?<br>- Was the length of follow up different across groups?	
Is the sample size large enough to detect a clinically significant difference of 5% of more between groups in at least 1 primary outcome?	
Are the statistical methods adequate?	
Are any important growth outcomes missing from the results?	
Are results believable?	
Can the results be applied to the local population?	

Supplementary Table 5: Summary of articles with mention of parental SES and growth outcomes for which growth outcomes were not reported separately by parental socioeconomic status.

Reference	Setting	Study design	Population	Exclusion criteria	Measure of parental socioeconomic status	Growth outcomes measured	Reported association between parental socioeconomic status and growth outcomes
**Arad 1990**[1]	**Israel**: preterm infants treated at the NICU of the Hadassah University Hospital, a teaching hospital in Jerusalem.	Prospective cohort study of preterm infants followed up regularly over their first 2 years.	Preterm infants *(gestational age at birth not reported)* treated at the NICU with birthweight < 1500 g. n = 101.	Infants who were not brought to follow-up clinic on a regular basis, and those who did not receive a developmental assessment between 1-2 years of age.	Parental education, parental occupation and the material condition of the family were assessed by a social worker and summarised into a collective categorical measure of parental socioeconomic status: Good, Mediocre or Poor.	Weight, length and head circumference were measured at the follow-up clinic an average of 5 times in the first year and twice in the second year. Measurements were classified into one of 3 groups based on the percentiles: A. Above 20th percentile, B. Between 3rd and 20th percentile, C. Below 3rd percentile.	The authors report a significant association between body length at the time of last examination at the clinic (average corrected age of 16 months) and parental socioeconomic status in a multivariable regression analysis (p value and growth data used not reported). Other covariates in the model were not explicitly specified but included the clinical course in

							the intensive care unit; neurological complications in the unit; birthweight; length; development, weight and head circumference at the last examination; and the attitude of the parents to the baby.
**Ross 1983**[2]	**United States:** Preterm infants discharged from the Perinatology Center of the New York Hospital-Cornell Medical Center, a teaching hospital in New York City.	Prospective cohort study of preterm infants followed up regularly up to 12 months post-term.	Preterm infants admitted to the Perinatology Center from Mar 1978-Sep 1979 with birthweight < 1500 g. n = 86. Mean GA = 29 weeks (+/- 1.8 weeks).	None reported.	Hollingshead Scale, measuring socioeconomic status based on marital status, retired/employed status, educational attainment, and occupational prestige: class I (high) to class V (low).	Weight, crown-heel length and occipitofrontal head circumference measured at the follow-up clinic at 1, 3, 6, 9 and 12 months post-term.	Comparison using t tests did not show any relationship between parental socioeconomic status and length, weight or head circumference at 12 months post-term (results of t tests and growth data used were not reported).
**Ross 1985**[3]	**United States:** Preterm infants discharged from the Perinatology Center of the New York Hospital-Cornell Medical	Prospective cohort study of preterm infants followed up at 3 to 4 years of age following their assessment at 12 months post-term.	Preterm infants admitted to the Perinatology Center from Mar 1978-Sep 1979 with birthweight < 1500 g. n = 79. Mean GA = 29 weeks (+/- 1.8 weeks).	Infants who did not return for follow-up at 3 to 4 years of age.	Hollingshead Scale.	Weight, crown-heel length and head circumference measured at the 3 year follow-up clinic.	Analysis of covariance showed that parental socioeconomic status was significantly related to head circumference at 3 years of age (p

	Center, a teaching hospital in New York City.						< 0.05, growth data used were not reported). The other covariates in the model were height and head circumference at 3 years; neurological status at 3 years; infant sex; paternal stature; and maternal stature.
**Hack 1984**[4]	**United States:** Preterm infants discharged from the NICU at Rainbow Babies and Children's Hospital, a teaching hospital in Cleveland.	Prospective cohort study of very low birthweight infants followed up over their first 3 years.	Small for gestational age and appropriate for gestational age (AGA) infants with birthweight < 1500g admitted to the NICU during 1977 and 1978.<br>AGA infants (n = 147):<br>Mean GA = 29 weeks.<br>GA range = 26-33 weeks.<br>SGA infants (n = 35):<br>Mean GA = 32 weeks.<br>GA range = 26-36 weeks.	Infants who were unavailable for follow-up, infants with incomplete follow-up data, infants who died during their first or second year of life. Although not specified as exclusion criteria, the authors report no infants had major congenital malformations or intrauterine infections diagnosed before 8 months of age (it is unclear if this was corrected for gestational age).	1. Hollingshead Scale.<br>2. Maternal education: % with education less than high school.	Weight, height and head circumference were measured at the follow-up clinic at 40 weeks (term date), and at 8, 21, and 33 months corrected age.	For the SGA infants, correlation analysis using Pearson's correlation coefficient showed a significant positive correlation between Hollingshead social class V (lowest stratum) and weight < -2 SD below the mean at 21 months corrected age (r = 0.393). The growth data used for this analysis were not reported.

**Hack 1986**[5]	**United States:** Preterm infants discharged from the NICU at Rainbow Babies and Children's Hospital, a teaching hospital in Cleveland.	Prospective cohort study of very low birthweight infants followed up over their first 3 years.	Appropriate for gestational age (AGA) infants with birthweight < 1500g admitted to the NICU during 1977 and 1978. n = 139.<br>Mean GA = 29 weeks (+/- 1.9 weeks).	As above, and infants who were small for gestational age.	1. Hollingshead Scale.<br>2. Maternal education: % with education less than high school.	Weight, height and head circumference were measured at the follow-up clinic at 40 weeks (term date), and at 8, 21, and 33 months corrected age. Growth classified as normal or subnormal (< -2 SD) for age separately for weight, height and head circumference at each follow-up.	A multivariable regression analysis showed that Hollingshead social class did not affect the probability of an infant being classified as subnormal for head circumference or weight at 8 months corrected age (growth data used were not reported). The other covariates in the model were IQ at 3 years; Hobel neonatal risk score; neurological impairment (assessed at 20 and 33 months); and infant race.
**Hack 2003**[6]	**United States:** Preterm infants discharged from the NICU at Rainbow Babies and Children's Hospital, a teaching hospital in Cleveland.	Prospective cohort study of very low birthweight infants followed up until 20 years of age.	Very low birthweight (birthweight < 1500g) infants with weight and height measures at 20 years. n = 195.<br>Mean GA:<br>Males: 29.6 weeks (+/- 2.2 weeks)	Participants with neurosensory impairments, or Liddle's syndrome. Participants who were pregnant at the 20-year follow-up. Participants with missing growth	Maternal education at child's eighth year, coded as:<br>1. < High school<br>2. High school<br>3. > High school	Weight and length or height were measured at birth, 40 weeks postmenstrual age, 8 months, 20 months, 8 years corrected age and 20 years postnatal age. Weight and length or height z-scores were	A multivariable linear regression analysis showed no significant association between maternal education (< High school vs High school) and weight, height and BMI z-scores at 20 years

			Females: 30.0 weeks (+/- 2.3 weeks)	measurements at 20 years.		calculated at all time points, and changes in z-scores were calculated between time points.	among very low birthweight females, but maternal education was significantly associated with the 20-year BMI z-score among males (p = 0.001, growth data used not reported). The other covariates in the model were: maternal race, maternal height, birthweight z-score, chronic illness at 20 years, and length of neonatal hospital stay. In a similar analysis among participants who were small for gestational age at birth, maternal education was significantly associated with 20-year BMI and height z-scores (p value and growth data not reported).
**Zellner 2000**[7]	**Germany:** Preterm infants	Retrospective cohort study of	Preterm infants with birthweight <	Children with incomplete data	1. Parental education, coded as:	At age 5 to 9.5 years, height,	In a multivariable linear regression

	from the Thuringian-Saxon area (catchment areas of the Clinic for Pediatric and Adolescent Medicine in Jena, the Infant Clinic in Chemnitz and the Clinic for Pediatric and Adolescent Medicine in Erfurt).	ex-preterm infants recruited at 5 to 9.5 years.	1500 g or GA < 32 weeks. n = 128.<br>Male: (n = 75)<br>Median GA = 30 weeks (not normally distributed).<br>GA range = 26-36 weeks.<br>Female: (n = 53)<br>Mean GA = 30.4 weeks.<br>GA range = 25-35 weeks.	sets of biological and social variables.	a. < 8 years of school<br>b. 10 years of school<br>c. 12 years of school/High school graduation.<br>2. Parental professional qualification, coded as:<br>a. No professional qualification<br>b. Skilled worker qualification<br>c. University or technical college qualification.	weight, head circumference and BMI were measured in clinic. The SD score of these measures corrected for age was calculated.	analysis, maternal education was not significantly associated with height SD score of female children (growth data used were not reported). The other covariates in the model were paternal height; gestational age; and birthweight.
**Bozynski 1990**[8]	**United States:** Preterm infants admitted to an intensive care nursery (specific hospital or location not reported).	Prospective cohort study of preterm infants followed up until 12 months corrected age.	90 preterm Black infants with birthweight < 1200 g admitted to the intensive care nursery between Mar 1979-Jun 1983.	Infants who were small or large for gestational age.	Hollingshead scale, dichotomised:<br>1. High = classes I-III<br>2. Low = classes IV, V.	Weight measured at birth, at each even-numbered week until discharge, and during each follow-up visit at 4, 8 and 12 months corrected age.	In a multivariate regression model with parental socioeconomic status as the only explanatory variable, parental socioeconomic status did not explain the variance in infants' growth over the study period (growth data used were not reported) .
**Liu 2019**[9]	**China:** Preterm infants from the Child Health Care Departments of 5 Women's	Retrospective multicentre cohort study of preterm infants recruited at 1 to 3.5 years.	Preterm infants with gestational ages as follows. n = 309.<br>- Over 34 weeks:	Infants with congenital malformations. Infants with severe disease, e.g. cerebral palsy,	1. Caregiver education, coded as:<br>a. Junior middle school education<br>b. Senior middle school education	Weight measurements at birth and 12 months actual age were collected. Weight was	A multivariable logistic regression analysis showed that caregivers' education was

	and Children's Hospitals in Chengdu (province of Sichuan).		67.3% (n = 208).<br>- 32-34 weeks: 19.1% (n = 59).<br>- Below 32 weeks: 13.6% (n = 42).	chronic nephritis, metastatic liver disease, leukaemia. Uncooperative caregivers, participant withdrawal from the study, caregivers lacking normal language expression and reading comprehension ability.	c. Junior college education<br>d. Undergraduate education<br>e. Graduate-level education and above.<br>2. Monthly average family income.	measured at the follow-up visit between 1 and 3.5 years. Catch-up growth was defined as body weight reaching the 10th percentile for corrected age in SGA children and the 25th percentile for corrected age in AGA children.	significantly associated with a reduced odds of catch-up growth, whereas monthly average family income was associated with an increased odds of catch-up growth (growth data used were not reported).
**Cho 2022**[10]	**United States:** Preterm infants from a NICU of a tertiary medical center in the southeastern US.	Prospective cohort study of preterm infants followed up until 40 weeks' gestational age.	Convenience sample of mothers of very preterm infants. n = 88 dyads (each dyad had one infant). Mean GA = 28.6 weeks.<br>Range = 24.2 – 33.5 weeks.	Mothers younger than 15 years. Mothers who did not speak English or were not the primary caregiver of the neonate. Mothers who were dependent on narcotics or other recreational drugs, were HIV positive, or had a documented serious medical or psychological problem (e.g. cancer, postpartum psychosis). With twins (n = 4 pairs), only one infant was included in the analysis.	1. Maternal education, coded as:<br>a. High school or less<br>b. Greater than high school diploma.<br>2. Type of health insurance: private insurance, yes or no.	Weight, length and head circumference were measured at birth and 40 weeks' gestational age.	A general linear mixed model showed that a maternal education level of high school or level, and having private insurance were not significantly associated with neonatal growth in length from birth to 40 weeks' gestational age (growth data used were not reported). The other covariates in the model were maternal age; maternal marital status, Black

							maternal race, maternal BMI, pregnancy complications, labor complications, gravida, parity and vaginal delivery mode.
**Lequien 1986[11]**	**France:** Preterm infants from the Lille University Hospital's Neonatology Department, a teaching hospital in Lille.	Retrospective cohort study of preterm infants discharged from Nov 1982 to Mar 1983.	Preterm infants discharged from the Neonatology Department from Nov 1982 to Mar 1983. n = 182. Mean GA = 33.3 weeks (+/- 2.2 weeks).	Not reported.	Professional qualification (parent not specified), according to the 1977 classification of the French National Institute of Statistics and Economic Studies (INSEE).	Infants were followed up in the immediate days following discharge, and a minimum of 4 times during their first year. The authors do not state that weight was measured at these appointments, but weights for infants have been reported up to 1 year of age.	No association was found between the infants' weight at discharge (measured at 39.7 weeks +/- 2.1 weeks) and socioeconomic status. The growth data and statistical methods used were not reported.
**Connors 1999[12]**	**Australia:** Preterm infants form the Mater Mothers' Hospital, a private tertiary hospital in Brisbane.	Prospective cohort study of extremely low birthweight infants followed up at 2 years corrected age.	Extremely low birthweight (birthweight < 1000 g) infants discharged alive from Jan 1987 to Dec 1992. n = 198.<br>Mean GA = 27 weeks (+/- 2.2 weeks).	Infants not assessed between 18 and 30 months of age, infants who were transferred interstate for whom limited information was available. Infant death.	Material education, coded as:<br>1. < Grade 10<br>2. Grade 12<br>3. University/Technical and Further Education	Weight at 2 years (range 18 – 30 months), classified as:<br>1. < 3rd centile<br>2. 3rd to 9th centile<br>3. $\geq$ 10th centile	No significant association was found between weight < 10th centile at 2 years and maternal level of education (growth data used were not reported). The statistical method used is not reported, but the

							authors report use of the squared statistic for other analyses of nominal data.
**Kitchen 1980**[13]	**Australia:** Preterm infants born at The Royal Women's Hospital, a teaching hospital in Melbourne.	Prospective cohort study of very low birthweight infants followed up at 2, 6 and 8 years corrected age.	Very low birthweight (birthweight 1000 – 1500g) inborn infants born between Feb 1996 and Mar 1970 who were long-term survivors. n = 158. GA at birth not reported.<br>Mean GA at discharge or at death of infants in the original cohort (n = 238) was 30.50 weeks or less.	Infants with "gross malformations", hydrops fetalis, or malformations requiring major surgery. Infants who died in the labour ward, and infants in whom respirations were not established on arrival in the nursery.	Congalton 7-point scale of social class based on father's occupation, developed in Australia.	Weight, height and head circumference measured at 8 years corrected age, classified as:<br>1. $< 10^{th}$ percentile<br>2. $10\text{-}49^{th}$ percentile<br>3. $\geq 50^{th}$ percentile	There was no significant correlation between social class and weight, height or head circumference $< 10^{th}$ percentile at 8 years corrected age (all Pearson correlation coefficients had $p > 0.05$, growth data used not reported).
**Boo 2007**[14]	**Malaysia:** Preterm infants from the NICU of Hospital Universiti Kebangsaan Malaysia, a tertiary teaching hospital in Kuala Lumpur.	Randomised controlled trial comparing weight gain, head growth and breastfeeding rates in very low birthweight infants with and without exposure to a short duration of skin-to-skin contact while in the NICU.	Very low birthweight (birthweight < 1501g) infants in a stable condition after recovery from "major adaptation problems to extrauterine life"; nursed in a closed incubator; not requiring ventilatory support apart from nasal CPAP; on $FiO_2 \leq$	Lethal or major malformations, severe perinatal asphyxia with evidence of hypoxic ischaemic encephalopathy, transfer to another hospital, abandoned by parents, lack of consent to participate in skin-to-skin contact from at least one parent or guardian.	1. Maternal education, recorded as:<br>a. Total duration in years.<br>b. Mothers with and without university (tertiary) education.<br>2. Maternal occupation, recorded as mothers who were and were not housewives.<br>3. Monthly household income, coded as:<br>a. RM < 2501<br>b. RM $\geq$ 2501	Weight and head circumference measured at discharge.<br>Mean daily weight gain (g/day) and weekly increase in head circumference (cm/week) calculated between enrolment into study and discharge.	A logistic regression analysis showed that neither the number of years of maternal education, nor maternal university education, were associated with weekly increase in head circumference (growth data used not reported).

			0.3 or a O_2 flow rate of < 0.2 L/min via nasal prongs; and tolerating enteral feeds constituting $\geq$ 50% of the required fluid volume. n = 126.<br>Mean GA:<br>Intervention group: 31 weeks.<br>Control group: 30.4 weeks.				The other covariates in the model were: exposure to skin-to-skin contact, head circumference at the time of enrolment, postmenstrual age at delivery, postmenstrual age of enrolment, maternal parity and receiving expressed breast milk at the time of enrolment or during the intervention.
**Saigal 2001**[15]	**Canada:** Preterm infants born in central-west Ontario.	Prospective cohort study of extremely low birthweight infants followed from birth to adolescence (12 – 16 years unadjusted age).	Extremely low birthweight (birthweight 501 – 1000g) born between 1977 and 1982 to residents in a geographically defined region in central-west Ontario. n = 154 in total, n = 132 included in regression analysis investigating association between parental SES and growth.	None reported.	Hollingshead scale, classified as:<br>1. Upper = classes I, II<br>2. Middle = class III<br>3. Lower = classes IV, V	Height, weight and head circumference were measured at 8 years unadjusted age and adolescence (12 – 16 years unadjusted age). BMI, height-for-age z-scores and weight-for-age z-scores were calculated.	A linear regression analysis of the extremely low birthweight cohort found that socioeconomic status was not significantly associated with height or weight at adolescence. The other covariates in the model were age, sex, maternal and paternal height or weight (depending on the

			Mean GA of whole cohort = 27 weeks (+/- 2 weeks).				growth outcome being considered), gestational age and birthweight (growth data used not reported).
**Dusick 2003[16]**	**United States:** Preterm infants cared born at NICHD Neonatal Research Network centers across the US.	Prospective cohort of extremely low birthweight infants followed up at 18 to 22 months corrected age.	Extremely low birthweight (birthweight 501 to 1000g) survivors born between Jan 1993 and Dec 1994 within the NICHD Neonatal Research Network with anthropometric measures at 18 to 22 months corrected age. n = 1151.<br>GA not reported, but first follow-up occurred at 36 weeks postmenstrual age.	None reported.	Primary caregiver with less than a high school degree (dichotomous variable).	Weight measured at 36 weeks postmenstrual age. Weight, length and head circumference measured at 18 to 22 months corrected age, classified as:<br>1. $\leq 10^{th}$ percentile.<br>2. $10^{th} – 90^{th}$ percentile.<br>3. $> 90^{th}$ percentile.<br>Weight-length ratio (weight/length) calculated at 18 to 22 months; poor growth was defined as weight-length ratio $\leq 10^{th}$ percentile.	A univariate analysis showed that the primary caregiver's education level was not associated with poor growth (growth data used not reported).
**Reuner 2009[17]**	**Germany:** Preterm infants born at the Department of Neonatology in	Prospective cohort of preterm infants followed up at 17 years of age.	Preterm (GA < 37 weeks), low birthweight (birthweight < 2500g) infants	Infants with inborn or acquired causes of developmental problems, significant	1. Parental educational level.<br>2. Parental Magnitude Prestige Score, calculated from	Self-reported weight and height at 17 years of age, obtained during a structured	A t-test showed no significant difference in the parental Magnitude

	the University Hospital of Heidelberg, a teaching hospital in Heidelberg.		born between Jul 1986 and Jun 1987 at the Department of Neonatology. n = 65.<br>Mean GA = 33.4 weeks (+/- 2.1 weeks)	handicaps after birth, significant postnatal risks (e.g. prolonged ventilation, neonatal seizures, signs of increased cerebral irritability, neonatal sepsis).<br>Infants with birthweight < 1000g or > 2500g.<br>Infants without German as the native language.	schooling and professional qualifications, professional status, and income.	telephone interview. Height and weight percentiles and BMI were calculated.<br>Length/height percentiles at 20 months and 7 years are mentioned, but it is not reported whether these measurements were self-reported or measured by researchers.<br>All infants with length/height < 10th percentile at 20 months, 7 and 17 years were classified as continuously small (CS-LBW). The other very low birthweight infants were classified as variable growth (VG-LBW).	Prestige Score between LBW infants who were continuously small and those who had variable growth.

1 Arad, I. & Netzer, D. [Growth and Development of Very-Low-Birth-Weight Infants]. *Harefuah* **118**, 1-5 (1990).

2 Ross, G., Krauss, A. N. & Auld, P. A. Growth Achievement in Low-Birth-Weight Premature Infants: Relationship to Neurobehavioral Outcome at One Year. *J Pediatr* **103**, 105-108 (1983).

3 Ross, G., Lipper, E. G. & Auld, P. A. Physical Growth and Developmental Outcome in Very Low Birth Weight Premature Infants at 3 Years of Age. *J Pediatr* **107**, 284-286 (1985).

4 Hack, M., Merkatz, I. R., McGrath, S. K., Jones, P. K. & Fanaroff, A. A. Catch-up Growth in Very-Low-Birth-Weight Infants. Clinical Correlates. *Am J Dis Child* **138**, 370-375 (1984).
5 Hack, M. & Breslau, N. Very Low Birth Weight Infants: Effects of Brain Growth During Infancy on Intelligence Quotient at 3 Years of Age. *Pediatrics* **77**, 196-202 (1986).
6 Hack, M. et al. Growth of Very Low Birth Weight Infants to Age 20 Years. *Pediatrics* **112**, e30-38 (2003).
7 Zellner, K. & Kromeyer-Hauschild, K. Somatic Development of Children Born with Very Low Birth Weight (< 1500 G) and/or Born Very Premature (< 32 Weeks of Gestation). *Monatsschr Kinderheilkd* **148**, 131-137 (2000).
8 Bozynski, M. E. et al. Bronchopulmonary Dysplasia and Postnatal Growth in Extremely Premature Black Infants. *Early Hum Dev* **21**, 83-92 (1990).
9 Liu, X. et al. Factors Affecting the Catch-up Growth of Preterm Infants after Discharge in China: A Multicenter Study Based on the Health Belief Model. *Italian Journal of Pediatrics* **45**, 87 (2019).
10 Cho, J., Chien, L. C. & Holditch-Davis, D. Sociodemographic and Biological Factors of Health Disparities of Mothers and Their Very Low Birth-Weight Infants. *Adv Neonatal Care* **22**, E169-e181 (2022).
11 Lequien, P. et al. [Discharge to the Family Milieu of Low Birth Weight Children. Analysis of an Experience with "Early Discharge" in a Neonatology Unit]. *Arch Fr Pediatr* **43**, 471-474 (1986).
12 Connors, J. M. et al. The Influence of Growth on Development Outcome in Extremely Low Birthweight Infants at 2 Years of Age. *J Paediatr Child Health* **35**, 37-41 (1999).
13 Kitchen, W. H., McDougall, A. B. & Naylor, F. D. A Longitudinal Study of Very Low-Birthweight Infants. Iii: Distance Growth at Eight Years of Age. *Dev Med Child Neurol* **22**, 163-171 (1980).
14 Boo, N. Y. & Jamli, F. M. Short Duration of Skin-to-Skin Contact: Effects on Growth and Breastfeeding. *J Paediatr Child Health* **43**, 831-836 (2007).
15 Saigal, S., Stoskopf, B. L., Streiner, D. L. & Burrows, E. Physical Growth and Current Health Status of Infants Who Were of Extremely Low Birth Weight and Controls at Adolescence. *Pediatrics* **108**, 407-415 (2001).
16 Dusick, A. M., Poindexter, B. B., Ehrenkranz, R. A. & Lemons, J. A. Growth Failure in the Preterm Infant: Can We Catch Up? *Semin Perinatol* **27**, 302-310 (2003).
17 Reuner, G., Hassenpflug, A., Pietz, J. & Philippi, H. Long-Term Development of Low-Risk Low Birth Weight Preterm Born Infants: Neurodevelopmental Aspects from Childhood to Late Adolescence. *Early Hum Dev* **85**, 409-413 (2009).
